# CRY2 interacts with CIS1 to regulate thermosensory flowering via FLM alternative splicing

**DOI:** 10.1038/s41467-022-34886-2

**Published:** 2022-11-17

**Authors:** Zhiwei Zhao, Craig Dent, Huafeng Liang, Junqing Lv, Guandong Shang, Yawen Liu, Fan Feng, Fei Wang, Junhong Pang, Xu Li, Libang Ma, Bing Li, Sridevi Sureshkumar, Jia-Wei Wang, Sureshkumar Balasubramanian, Hongtao Liu

**Affiliations:** 1 National Key Laboratory of Plant Molecular Genetics, CAS Center for Excellence in Molecular Plant Sciences, Institute of Plant Physiology and Ecology, Chinese Academy of Sciences, 200032 Shanghai, China; 2 University of Chinese Academy of Sciences, 200032 Shanghai, China; 3 School of Biological Sciences, Monash University, Clayton Campus, VIC 3800 Australia; 4 College of Life Sciences, Henan University, 475001 Kaifeng, China; 5 College of Life Sciences, Hebei Normal University, 050024 Shijiazhuang, China

**Keywords:** Light responses, Plant signalling, Plant development, Flowering

## Abstract

Cryptochromes (CRYs) are evolutionarily conserved photolyase-like photoreceptors found in almost all species, including mammals. CRYs regulate transcription by modulating the activity of several transcription factors, but whether and how they affect pre-mRNA processing are unknown. Photoperiod and temperature are closely associated seasonal cues that influence reproductive timing in plants. CRYs mediate photoperiod-responsive floral initiation, but it is largely unknown whether and how they are also involved in thermosensory flowering. We establish here that blue light and CRY2 play critical roles in thermosensory flowering in *Arabidopsis thaliana* by regulating RNA alternative splicing (AS) to affect protein expression and development. CRY2 INTERACTING SPLICING FACTOR 1 (CIS1) interacts with CRY2 in a blue light–dependent manner and promotes CRY2–mediated thermosensory flowering. Blue light, CRYs, and CISs affect transcriptome-wide AS profiles, including those of *FLOWERING LOCUS M* (*FLM*), which is critical for temperature modulation of flowering. Moreover, CIS1 binds to the *FLM* pre-mRNA to regulate its AS, while CRY2 regulates the RNA-binding activity of CIS1. Thus, blue light regulates thermosensory flowering via a CRY2–CIS1–FLM signaling pathway that links flowering responses to both light and ambient temperature.

## Abstract (web summary)

CRY2 mediates photoperiod-responsive floral initiation in Arabidopsis. Here the authors show that CRY2 also regulates temperature sensitive flowering and that CRY2 can interact with and regulate the splicing factor CIS1 to affect alternative splicing of transcripts that regulate flowering.

## Introduction

Light and temperature change in concert with seasonal cycles. Most plants flower at a certain time of year, indicating that they use light and temperature cues to coordinately regulate flowering. The photolyase-like blue-light receptors cryptochromes (CRYs) were initially discovered in Arabidopsis (*Arabidopsis thaliana*) and later widely identified in almost all species examined. The Arabidopsis genome encodes at least two CRYs, CRY1 and CRY2. The major function of CRY1 is to mediate blue light-dependent de-etiolation responses^1^, whereas CRY2 primarily participates in photoperiodic regulation of floral initiation, as evidenced by the very late flowering phenotype of *cry2* mutants in a long day (LD) but not short day (SD) conditions when plants are grown at the typical temperature of 22 °C^2^. Photoexcited CRY undergoes a series of biophysical and biochemical changes, including circular electron transfer, dimerization, phosphorylation, ubiquitination, and conformational changes to alter gene transcription at the transcriptional level and protein abundance at both transcriptional and posttranslational level^3–6^. CRY2 protein abundance is modulated not only by blue light but also by ambient temperatures^7^. CRYs may mediate photoperiodic control of floral initiation by at least three different mechanisms. First, CRYs directly modulate *FLOWERING LOCUS T (FT)* mRNA levels in response to blue light by interacting with the basic helix–loop–helix (bHLH) transcription factor CRY2 INTERACTING bHLH 1 (CIB1) and its homologs^8–11^. Second, CRY2 interacts with SUPPRESSOR OF PHYA1-105 1 (SPA1) to suppress the CONSTITUTIVE PHOTOMORPHOGENIC 1 (COP1)-dependent degradation of CONSTANS (CO)^12–14^, a major transcriptional regulator of photoperiodic floral initiation^15,16^. Third, CRYs contribute to light entrainment of the circadian clock^17^, which affects the expression pattern of the clock-controlled gene *CO*. Moreover, CRY2, CIB1, and CO form a protein complex in response to blue light that regulates *FT* expression and photoperiodic flowering^10^. Whether or how CRYs affect other steps besides transcription and protein stability is a major outstanding question^18^.

Pre-mRNA splicing involves the removal of introns from pre-mRNAs. Introns can be constitutively or alternatively spliced. Alternative splicing (AS) may lead to intron removal or retention or may engage alternative 5’ and/or 3’ splice sites (SSs), producing multiple transcripts from a single gene. AS is a critical posttranscriptional event that adjusts transcript abundance and expands transcriptome diversity without increasing gene number^19,20^. Large ribonucleoprotein complexes called spliceosomes are critical for pre-mRNA splicing^19,20^. Five small nuclear ribonucleoprotein (snRNP) complexes (U1, U2, U3, U5, and U6) recognize and sequentially assemble onto each intron to catalyze its removal^19^. The red/far-red light photoreceptor phytochrome B (phyB) regulates photomorphogenesis and light-mediated development by modulating pre-mRNA splicing of many light signaling and circadian clock genes through SPLICING FACTOR FOR PHYTOCHROME SIGNALING (SFPS)^21,22^. CRYs have not been reported to affect AS.

Variation in ambient growth temperature also dramatically affects the time of flowering; for example, high ambient temperatures (27 °C instead of 22 °C) promote flowering under SD conditions, largely substituting for longer photoperiods in inducing flowering. The MADS-domain proteins FLOWERING LOCUS M (FLM) and SHORT VEGETATIVE PHASE (SVP) participate in ambient temperature-regulated flowering^23,24^. Ambient temperature-controlled AS of *FLM* and SVP protein stability is critical for temperature-regulated flowering. *FLM* pre-mRNA undergoes differential AS in response to changes in ambient temperature^23–25^. The level of *FLM β* transcripts, an alternatively spliced isoform of *FLM*, increases at lower temperatures to repress flowering^25–27^. The encoded protein FLM β interacts with SVP, after which the resulting FLM β–SVP complex binds to DNA to repress flowering. However, SVP becomes degraded at high temperatures, and *FLM β* transcript level decreases, reducing the abundance of the FLM β–SVP complex and alleviating floral repression^26,27^. The late flowering phenotype of *cry2* mutants is more pronounced at 16 °C than at 22 °C under LD conditions^28^, but whether and how CRY2 regulates thermosensory flowering remains largely unknown.

Here we show that CRY2 integrates light and temperature signals to regulate flowering. CRY2 interacts with CRY2 INTERACTING SPLICING FACTOR 1 (CIS1) in a blue light-dependent manner to promote flowering. Blue light, CRYs and CISs affect global AS profiles, including AS of *FLM*, which is critical for temperature-regulated flowering. Moreover, CIS1 binds to the *FLM* pre-mRNA to regulate its AS and thus flowering, with CRY2 regulating the RNA-binding activity of CIS1. Our results expose a new mechanism that controls flowering in response to changes in light and ambient temperatures, whereby blue light regulates thermosensory flowering via the CRY2–CIS1–FLM signaling pathway.

## Results

### CRY2 integrates light and temperature signals to regulate flowering and interacts with CIS1 in a blue light-dependent manner

**Fig. 1:**
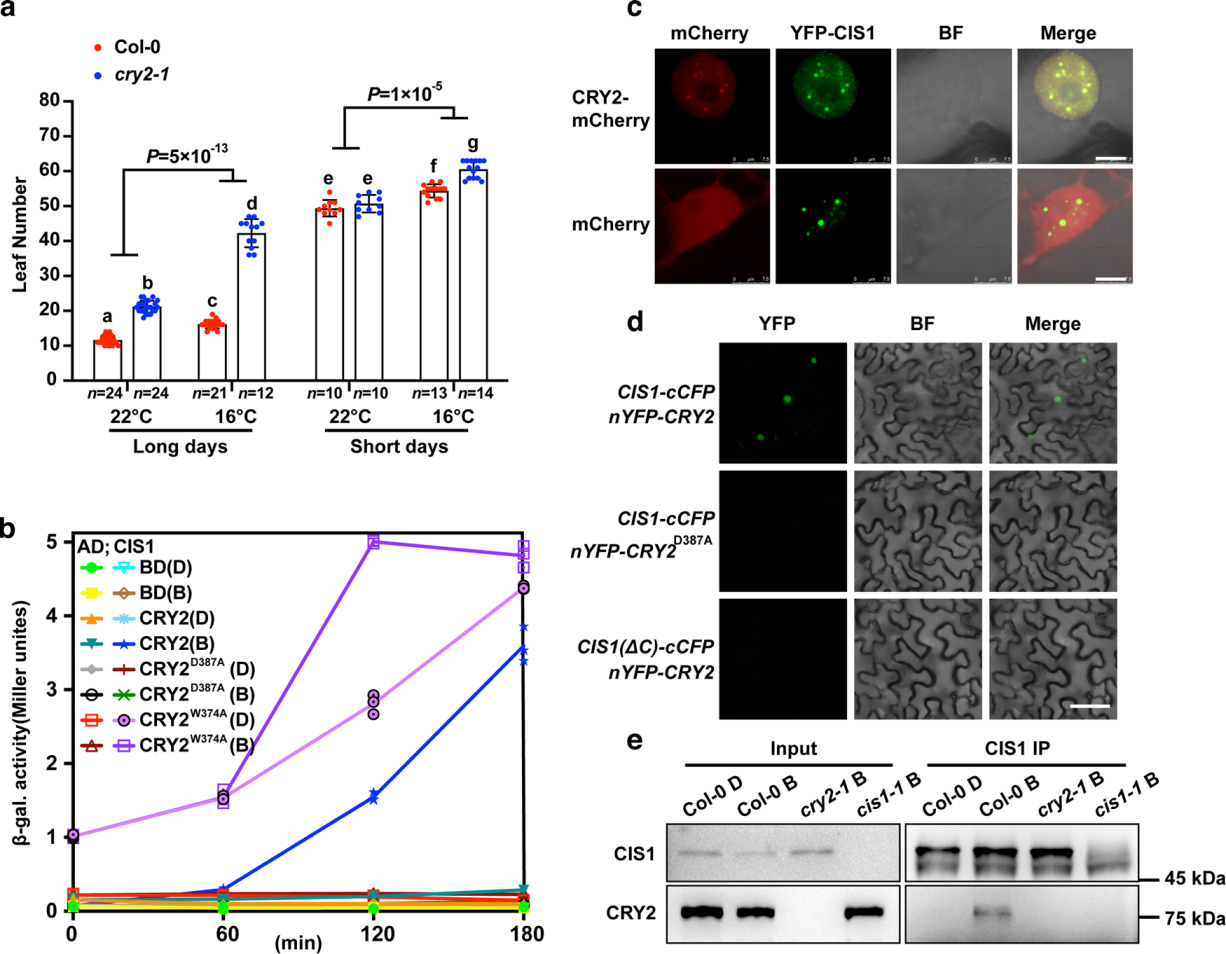
CRY2 integrates light and temperature signals to regulate flowering and CRY2 interacts with CIS1 in a blue light-dependent manner. **a** Number of rosette leaves at the time of flowering for *cry2-1* at 22 and 16 °C under LD (16-h light, 8-h dark) or SD (8-h light, 16-h dark) photoperiods. Blue and red circles indicate the data from individual plants. Error bars represent standard deviation (s.d., *n* ≥ 10). Lowercase letters indicate statistically significant differences, as determined by one-way ANOVA with Tukey’s multiple comparisons test (*P* < 0.05). Two-sided Student’s *t*-test was used to calculate *P* values between groups. **b** β-gal assays of yeast cells grown at –LT medium 28 °C in darkness (D) or exposed to blue light (B, 30 μmol m^–2^ s^–1^) for the indicated times. Three biological replicates are listed. CRY2^D387A^ is a site-specific mutant of CRY2 that cannot be activated by blue light. CRY2^W374A^ is a constitutively active site-specific mutant of CRY2. **c** Co-localization of CRY2 and CIS1 in nuclear speckles in *N. benthamiana* leaves. mCherry served as a negative control. BF, brightfield. Merge and overlay the YFP, and brightfield images. Scale bar = 5 μm. **d** BiFC assay showing in vivo protein interaction between CRY2 and CIS1. *CIS1(ΔC)-cCFP* and *nYFP-CRY2*^*D387A*^ were used as negative controls. *N. benthamiana* was co-infiltrated with the indicated constructs. Scale bar = 20 μm. **e** Co-IP assays showing that CRY2 interacts with CIS1 in a blue light-dependent manner in plant cells. 22 °C LD-grown 7-day-old Col-0, *cry2-1*, and *cis1-1* seedlings were pretreated in darkness for 24 h, then treated with 25 μM MG132 in the dark for 2 h and exposed to blue light (B, 30 μmol m^–2^ s^–1^) for 20 min. Input: immunoblots showing the abundance of CIS1 and CRY2 in the total protein extracts. CIS1 immunoprecipitation (IP): IP products precipitated by the anti-CIS1 antibody. Total proteins (Input) or IP products of CIS1-beads (CIS1 IP) were probed in immunoblots with an anti-CIS1 or anti-CRY2 antibody. In **c**–**e**, three independent experiments were performed with similar results.

*cry2* mutants flower very late when grown in LD but not SD conditions at 22 °C, supporting the idea that CRY2 has a role in photoperiodic flowering^2^. We observed that *cry2* mutants flower normally under SD conditions at 22 °C but exhibited a delay in flowering at 16 °C (Fig. 1a), suggesting that CRY2 modulates flowering not only in LD conditions but also in SD conditions at lower ambient temperatures (16 °C). This finding together with the more severe late flowering phenotype of *cry2* mutants at 16 °C than at 22 °C under LD conditions^28^ (Fig. 1a and Supplementary Fig. 1a) led us to hypothesize that CRY2 influences ambient temperature-modulated flowering independently of photoperiod.

To address how CRY2 might influence ambient temperature-regulated flowering and how CRY2 affects other steps besides transcription and protein stability, we looked for proteins interacting with CRY2 by yeast two-hybrid assay and identified an uncharacterized SURP and G patch (SUGP) domain-containing protein (At3g52120) (Fig. 1b–e and Supplementary Fig. 1b–d). Since SUGP-domain proteins are RNA-binding proteins suggested to play a role in RNA splicing, we named this factor CRYPTOCHROME-INTERACTING SPLICING FACTOR 1 (CIS1). In yeast cells, CIS1 interacted with CRY2 when illuminated with blue light but not in darkness, as demonstrated by two different assays (Fig. 1b and Supplementary Fig. 1b). First, yeast cells exposed to 30 µmol m^–2^ s^–1^ blue light showed appreciable β-galactosidase (β-gal) reporter activity as early as after 60 min (Fig. 1b), but we measured no β-gal activity in cells maintained in darkness (Fig. 1b). Second, we failed to detect an interaction between CIS1 and the flavin-deficient CRY2 variant CRY2^D387A^^ 8^ (Fig. 1b and Supplementary Fig. 1b), whereas the constitutively active CRY2 variant CRY2^W374A^ ^29^ interacted with CIS1 under both blue light and darkness (Fig. 1b and Supplementary Fig. 1b), indicating that the CRY2–CIS1 interaction depends on the flavin chromophore of the CRY2 photoreceptor. In agreement, the N-terminal domain of CRY2 was sufficient for interaction and consisted of the chromophore-binding domain, while the C-terminal domain (G patch) of CIS1 was sufficient for interaction in yeast cells (Supplementary Fig. 1b–d).

CIS1 co-localized with CRY2 in the nucleus (Fig. 1c). In addition, CIS1 interacted with CRY2 in plant cells in bimolecular fluorescence complementation (BiFC) assays (Fig. 1d), as evidenced by the strong yellow fluorescent protein (YFP) fluorescence detected in the nuclei of *Nicotiana benthamiana* leaves transiently co-infiltrated with *CIS1-cCFP* and *nYFP-CRY2* constructs (Fig. 1d), in contrast to leaves co-infiltrated with *CIS1-cCFP* and *nYFP-CRY2*^*D387A*^ (expressing the flavin-deficient CRY2 variant), or *CIS1(ΔC)-cCFP* (producing the N terminus of CIS1 only) and *nYFP-CRY2* constructs. These results indicated that CIS1-cCFP and nYFP-CRY2 interact to reconstitute a functional fluorophore (Fig. 1d). As in yeast, CRY2^D387A^ did not interact with CIS1, in agreement with the observed dependence on the CRY2 chromophore for the CRY2–CIS1 interaction. We also detected an interaction between CIS1 and CRY2 by bimolecular luminescence complementation (BiLC) in plant cells, as determined by the strong luminescence of leaves co-infiltrated with *cLUC-CRY2* and *CIS1-nLUC* constructs (Supplementary Fig. 1e, f). Using BiLC assays, we totally identified 7 out of 15 G-patch-containing proteins in *Arabidopsis* as interacting partners of CRY2, including At1g63980, the closest homolog to CIS1, which we named CIS2 (Supplementary Fig. 1e, f).

To investigate whether the CRY2–CIS1 interaction is regulated by blue light in plant cells, we performed co-immunoprecipitation (co-IP) assays. We first pretreated seedlings with the proteasome inhibitor MG132 to block blue light-dependent CRY2 degradation^30^. We then exposed seedlings to blue light (30 μmol m^–2^ s^–1^ for 20 min) or kept them in darkness before conducting co-IP. CRY2 co-precipitated with CIS1 in the samples treated with blue light (Fig. 1e). By contrast, little CRY2 co-precipitated with CIS1 in the samples maintained in darkness (Fig. 1e). These results strongly suggested that blue light stimulates the accumulation of the CRY2–CIS1 complex in plant cells. We concluded that CRY2 interacts with CIS1 in response to blue light.

Phylogenetic analysis revealed a high degree of conservation among CIS1 orthologs during evolution (Supplementary Fig. 2a), as are CRYs. We, therefore, tested the interaction between human CRY2 (hCRY2) and human CIS1 (hCIS1) homologs to see whether the interaction between CRYs and G-patch domain-containing RNA-binding proteins are evolutionarily conserved between plants and mammals; of the 14 hCIS1 homologs tested, 4 interacted with hCRY2 in yeast cells, including the putative splicing factor SURP and G Patch Domain Containing 1 (SUGP1), which regulates cholesterol metabolism^31^ (Supplementary Fig. 2b).

### CIS1 promotes flowering in a CRY-dependent manner

We determined the expression pattern of *CIS1* to understand its biological roles by using reporter lines in which the CIS1 promoter drove the transcription of the reporter *gene* *β-GLUCURONIDASE*. We detected GUS staining in all seedling tissues as well as in mature leaves, roots, and flowers (Supplementary Fig. 3a). Blue light (30 µmol m^–2^ s^–1^) slightly induced the transcription of *CIS1* in the first 30 min of treatment, after which transcription decreased (Supplementary Fig. 3b), as shown by quantitative reverse transcription PCR (RT-qPCR). We also investigated CIS1 protein levels by generating transgenic plants constitutively accumulating epitope-tagged CIS1 (*35S:MYC-CIS1*). Blue light promoted the accumulation of CIS1 (Supplementary Fig. 3c). Conversely, transferring seedlings to darkness induced the degradation of CIS1 via the 26S proteasome, as treatment with the 26S proteasome inhibitor MG132 resulted in the accumulation of CIS1 in darkness (Supplementary Fig. 3c). Furthermore, CIS1 protein levels increased in response to blue light and decreased in the absence of blue light in both WT seedlings and the *cry1 cry2* double mutant (Supplementary Fig. 3d), which argued that neither CRY1 nor CRY2 contributes to the blue-light block of CIS1 degradation. CIS1 protein stability also appeared to be regulated by temperature. MYC-CIS1 abundance rose when seedlings were shifted from 16 to 22 °C and dropped when shifted from 22 to 16 °C. Treating seedlings with MG132 resulted in the strong accumulation of MYC1-CIS1 upon transfer from 22 to 16 °C, indicative of the involvement of the 26S proteasome (Supplementary Fig. 3e).

**Fig. 2:**
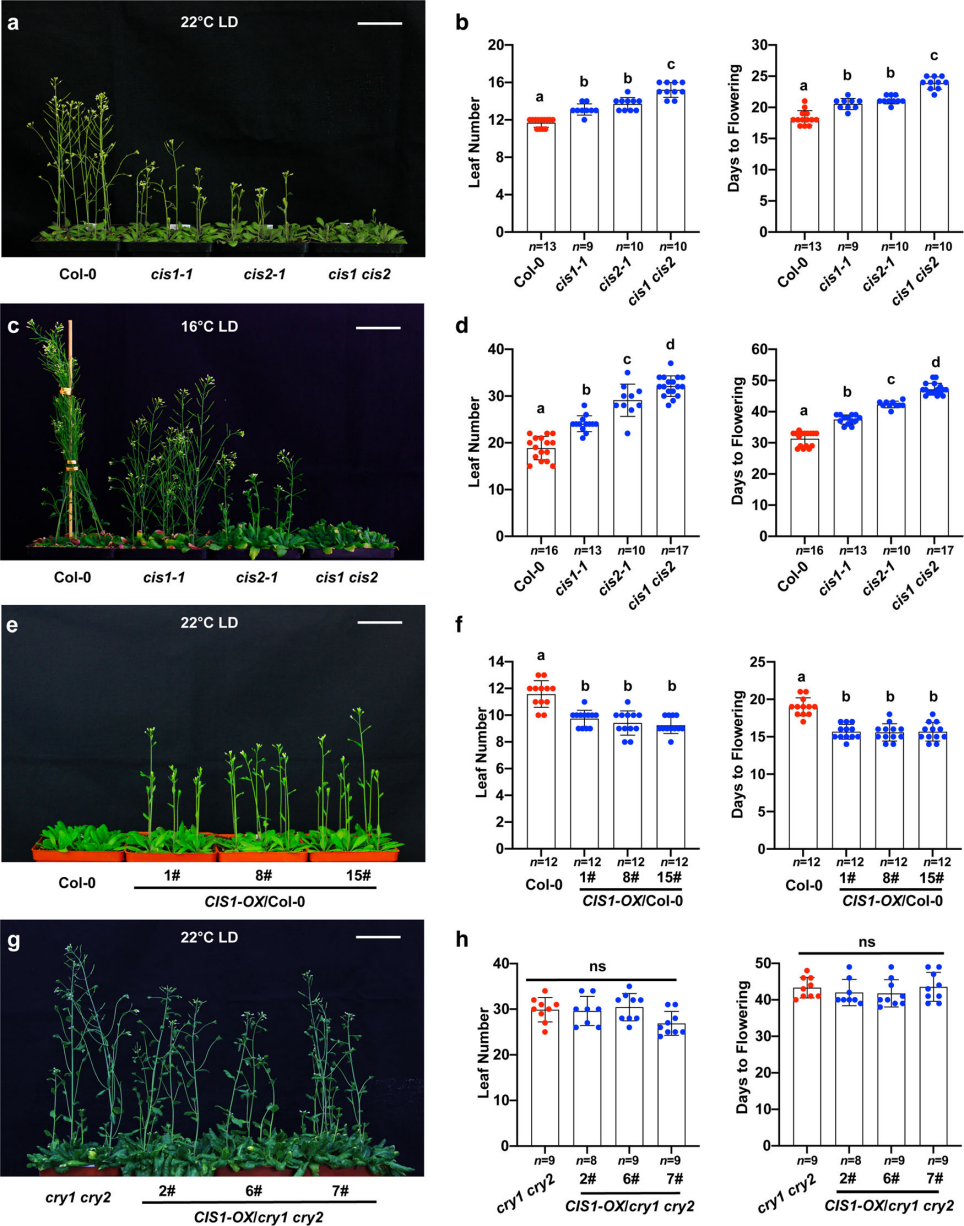
CIS1 promotes flowering in a CRY-dependent manner. **a**, **c** Flowering phenotype of *cis* mutants. Representative photographs of 27-day-old plants at 22 °C (**a**) or 55-day-old plants at 16 °C (**c**) of the indicated genotypes grown in LD conditions. **e**, **g** Flowering phenotype of *CIS1* overexpressing transgenic lines. Representative photographs of three independent 20-day-old (**e**) *CIS1-OX/*Col-0 or 55-day-old (**g**) *CIS1-OX/cry1 cry2* grown at 22 °C in LD conditions. Scale bars = 5 cm. **b**, **d**, **f**, and **h** Number of rosette leaves at the time of flowering and days to flowering of the indicated genotypes shown in **a** (for **b**), **c** (for **d**), **e** (for **f**), and **g** (for **h**). Blue and red circles indicate the data from individual plants. Error bars represent the s.d. For **b**, **d**, **f**, and **h**, lowercase letters indicate statistically significant differences, as determined by one-way ANOVA with Tukey’s multiple comparisons test (*P* < 0.05).

The *cis1-1* and/or *cis2-1* loss-of-function mutants showed a late-flowering phenotype at 22 °C in LD, LD blue light (30 µmol m^−2^ s^−1^), SD and at 16 °C in SD conditions (Fig. 2a, b and Supplementary Fig. 4a–h), whereas the *cis1 cis2* double mutant flowered later than the WT (Col-0) at 22 and 16 °C in both LD and SD conditions (Fig. 2a, b and Supplementary Fig. 4h). In LD conditions, the late flowering phenotypes of the *cis1-1*, *cis2-1*, and *cis1 cis2* mutants were more pronounced at 16 °C (mutant: WT ratios of 1.26, 1.43, and 1.76, respectively) than at 22 °C (mutant: WT ratios of 1.13, 1.17, and 1.3, respectively) (Fig. 2a–d and Supplementary Fig. 4i). The late flowering phenotype of the *cis1-1* mutant was complemented by the introduction of a *ProCIS1:CIS1-Flag* transgene (Supplementary Fig. 4j–m). Together, these results suggested that CIS1 and CIS2 are both involved in regulating thermosensory flowering.

Consistent with a role for CIS1 in floral initiation, transgenic plants overexpressing *CIS1* in the WT background flowered significantly earlier than the WT when grown at 22 °C in both LD and SD conditions (Fig. 2e, f, Supplementary Fig. 4n, o). We reasoned that if the function of CIS1 in promoting floral initiation is directly related to its physical interaction with CRY2, CIS1 activity should then be dependent on functional CRY2. To test this idea, we overexpressed *YFP-CIS1* in the *cry2-1* and *cry1 cry2* mutant backgrounds. *CIS1-OX/cry2-1* and *CIS1-OX /cry1 cry2* plants flowered at the same time as the *cry2-1* or *cry1 cry2* mutant at 22 or 16 °C in LD conditions (Fig. 2g, h and Supplementary Fig. 4p, q). YFP-CIS1 derived from the transgene accumulated to comparable levels in the Col-0 and *cry1 cry2* backgrounds, as demonstrated by immunoblot analysis with anti-GFP antibodies, indicating that the different effects of *CIS1* overexpression in the two different genetic backgrounds are not due to distinct CIS1 abundance (Supplementary Fig. 4r). We concluded that CIS1 function in promoting floral initiation depends on CRY2.

### Blue light and CRYs are involved in RNA splicing and share targets with CIS1

**Fig. 3:**
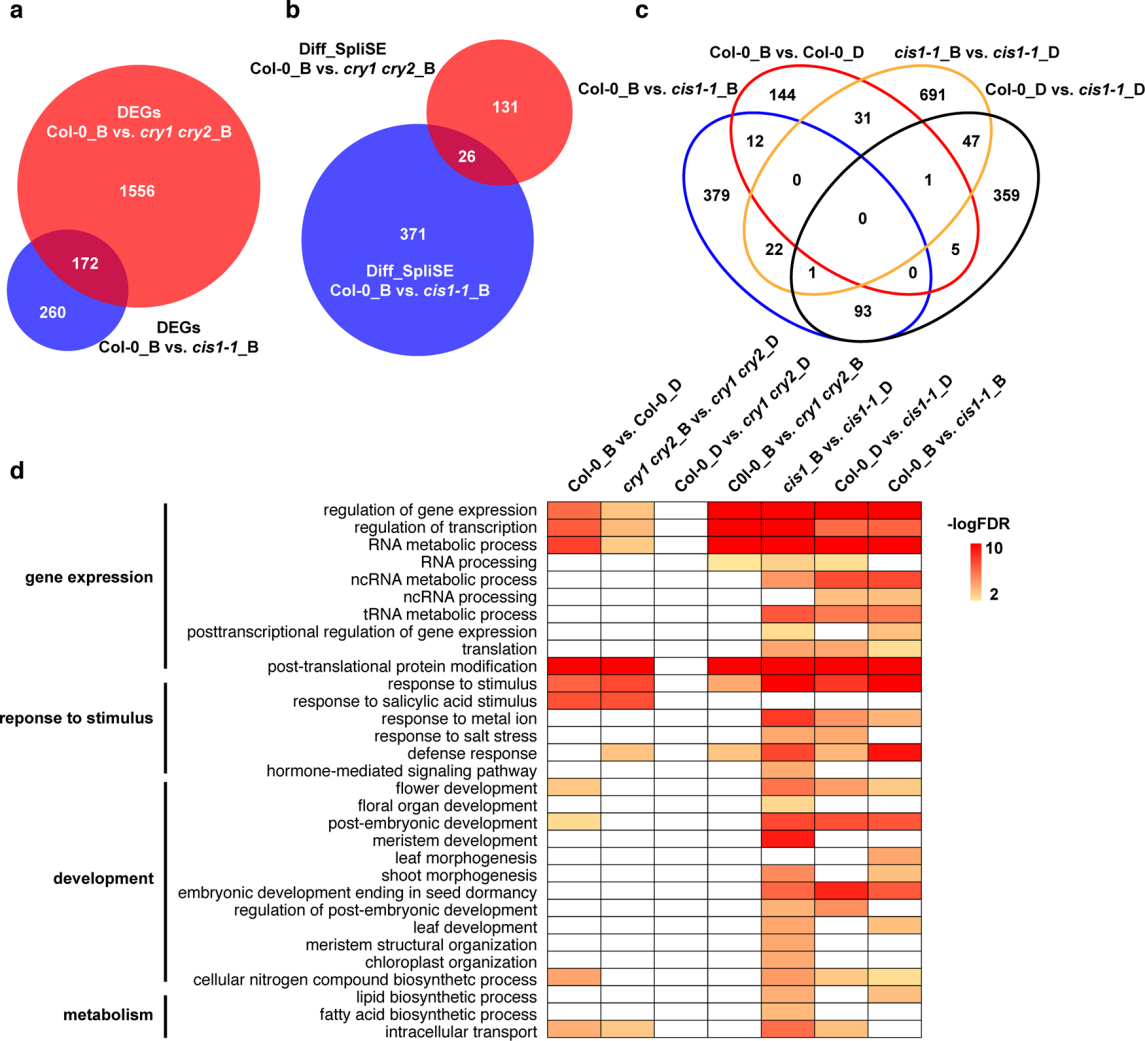
Blue light and CRYs are involved in RNA splicing and share targets with CIS1. **a**, **b** Venn diagram showing the overlap between sets of DEGs (**a**) and of differentially splice-site strength estimate genes (Diff SpliSE in **b**) with FDR < 0.01 in Col-0 versus the *cry1 cry2* double mutant in blue light (Col-0 B vs. *cry1 cry2* B) or Col-0 versus the *cis1-1* mutant in blue light (Col-0 B vs. *cis1-1* B) at 22 °C. **c** Venn diagram showing the overlap between sets of differentially splice-site strength estimate genes with FDR < 0.01 in Col-0 versus the *cis1-1* mutant in blue light (Col-0 B vs. *cis1* B) or Col-0 versus the *cis1-1* mutant in the dark (Col-0 D vs. *cis1-1* D), Col-0 B versus Col-0 D and *cis1-1*_B versus *cis1-1* D. **d** Overrepresented GO terms in the Diff SpliSE gene sets identified with FDR < 0.01 (−Log(FDR) > 2) were considered as significantly enriched.

Whether CRYs regulate RNA splicing is unknown. The CRY2-interacting protein CIS1 is a G-patch domain-containing RNA-binding protein that might be involved in pre-mRNA splicing, raising the possibility that CRY2 may regulate light-dependent RNA splicing and affect floral initiation. To test this hypothesis, we compared the transcriptomes of 4-day-old, etiolated Col-0, *cry1 cry2*, and *cis1-1* seedlings without or with a 3-h exposure to blue light (30 μmol m^–2^ s^–1^). Consistent with the roles of CRYs as blue-light photoreceptors, *cry1 cry2* mutant seedlings were associated with the highest number of differentially expressed genes (DEGs) compared to WT upon blue-light treatment (Fig. 3a, Supplementary Fig. 5a and Supplementary Data 1). About 40% of DEGs (172 of 432) in the *cis1-1* mutant were also differentially expressed in the *cry1 cry2* mutant specifically under blue-light conditions (hypergeometric probability *P* = 2.91 × 10^–54^), suggesting an overlapping role between CIS1 and CRY1/2 in blue light-dependent regulation of transcript levels. Nevertheless, the differences in the number of DEGs also pointed out that CRY1 and CRY2 are the primary determinants of blue light-dependent changes in gene expression.

Next, we analyzed splicing patterns using SpliSER, which estimates the strength of every splice site across the genome^32^. We detected more differentially spliced splice sites per gene in the *cis1-1* mutant (506 sites/415 genes in the dark and 507 sites/397 genes in blue light) compared to *cry1 cry2* (2 sites/2 genes in the dark and 171 sites/157 genes in blue light) (Fig. 3b, Supplementary Fig. 5b and Supplementary Data 2). Among the differentially spliced genes in *cis1-1*, about 25% (94 of ~400 genes) displayed an effect both in the dark and in blue light. In addition, heatmap representations of transcript levels showed that the splicing patterns were altered in the *cis1-1* mutant compared to the WT under both dark and light conditions, indicating a general light-independent role for CIS1 in splicing for these genes (Fig. 3c and Supplementary Fig. 5c–e). Among the differentially spliced genes in *cry1 cry2*, 17% (26/157 genes) were also differentially spliced in *cis1-1* (hypergeometric probability, *P* = 4.0 × 10^–21^, Fig. 3b), which suggested that the splicing changes mediated by CRY1 and CRY2 may partially depend on CIS1. There was little overlap between DEGs and differentially spliced genes in either *cis1-1* vs. WT or *cry1 cry2* vs. WT, which suggested that transcription and splicing are regulated in a largely independent manner, with CRY1/2 being a major player in regulating gene expression and CIS1 being a primary player in regulating splicing.

Gene Ontology (GO) enrichment analysis using the GO database (http://bioinfo.cau.edu.cn/agriGO/)^33^ identified several distinct processes associated with genes exhibiting blue light-regulated AS (Fig. 3d and Supplementary Data 3), including genes involved in blue light-regulated transcription, protein translation and stability, and response to external and endogenous stimuli through RNA AS. Furthermore, genes involved in the development of flowers, leaves, and shoots, and genes involved in lipid metabolism and fatty acid biosynthesis, were enriched in differentially spliced genes in response to blue light in *cis1-1* when compared to etiolated *cis1-1* seedlings, suggesting that CIS1 is required for normal plant development in response to blue light.

### Blue light and CRYs modulate *FLM* alternative splicing via CIS1

**Fig. 4:**
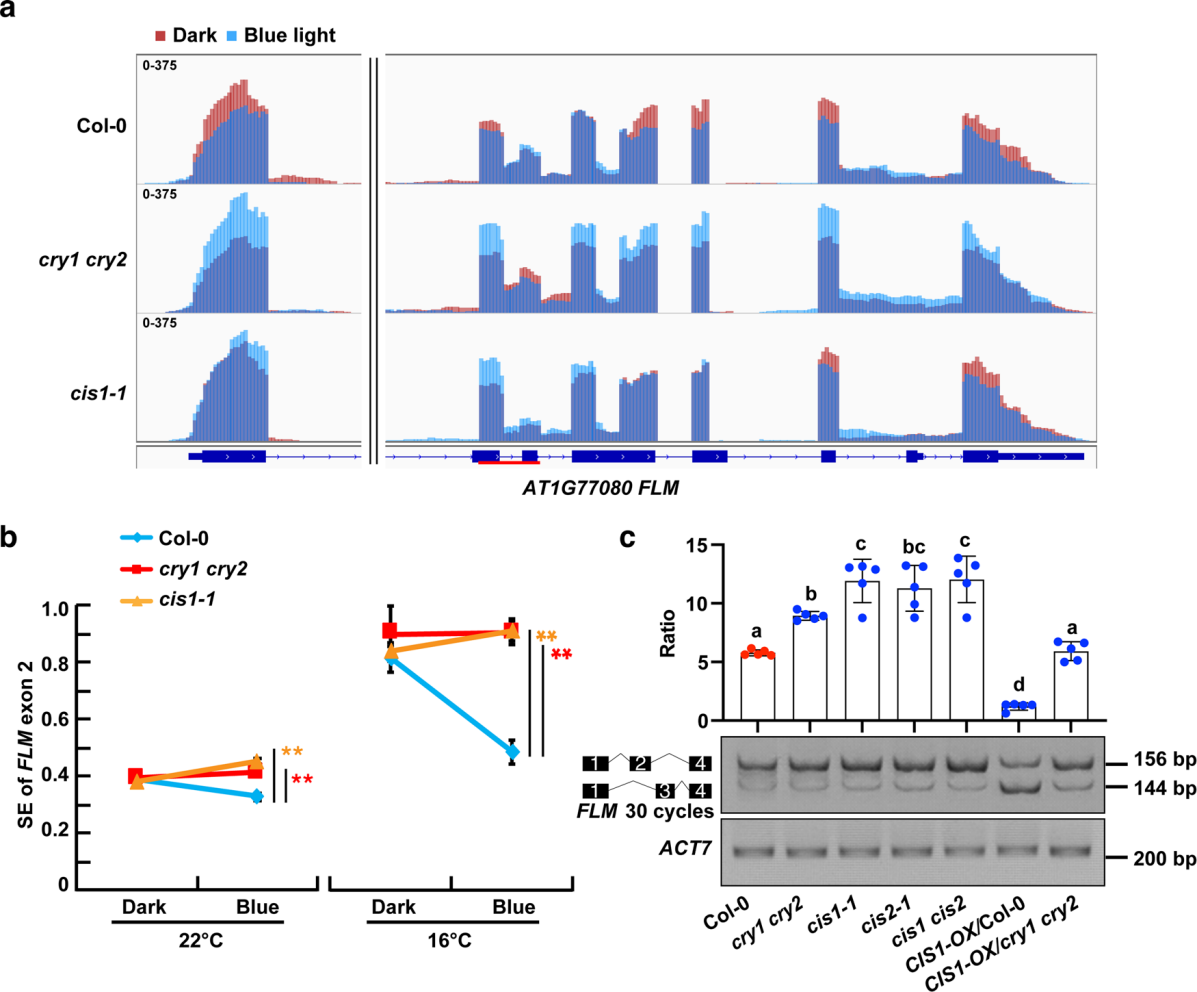
Blue light and CRYs modulate *FLM* AS via CIS1. **a** Integrative Genomics Viewer genome browser view showing AS of *FLM* in Col-0, *cry1 cry2*, and *cis1-1* at 22 °C as detected by transcriptome deep sequencing (RNA-seq). Dark red (Dark) and blue (Blue light, 30 μmol m^−2^ s^−1^ for 3 h) bars represent three biological replicates. The red line in the gene diagram indicates the position of AS events in *FLM*. **b** RT-qPCR validation blue light-regulated splice pattern of *FLM* in etiolated seedlings at 22 and 16 °C. Blue light-regulated splicing efficiency (SE) of *FLM* exon 2 was calculated as a proportion to total *FLM* transcripts. Error bars represent the s.d. of three biological replicates. The asterisks indicate a significant difference from Col-0 based on a two-sided Student’s *t*-test (**P < 0.01). **c** RT-PCR showing the AS pattern of *FLM* transcripts over exon 1–exon 2 and exon 1–exon 3 of the indicated genotypes 10-day-old seedlings grown at 22 °C in LD condition. Semi-quantitative splicing ratios of *FLM* exon 1–exon 2 to exon 1–exon 3 are shown as mean ± s.d. of five independent experiments. Red and blue circles indicate the data from individual plants. Lowercase letters indicate statistically significant differences, as determined by one-way ANOVA with Tukey’s multiple comparisons test (*P* < 0.05).

CIS1 is involved in pre-mRNA splicing, and we selected five genes showing AS in a blue light- and CIS1-dependent manner for independent verification using RT-qPCR (Supplementary Data 4, *P* < 0.05). RT-qPCR confirmed that CRYs and CIS1 mediated the blue light-regulated RNA splicing of multiple genes (*P* < 0.05) including *FLOWERING LOCUS M (FLM)* (Fig. 4a, b and Supplementary Fig. S6a–h). *FLM* pre-mRNA undergoes differential AS in response to changes in ambient temperature^23–25^. The levels of *FLM β* transcripts, one of the alternatively spliced isoforms of *FLM*, increases at lower temperatures to repress flowering^25–27^.

We observed that the levels of exon 2 (which is present mainly in *FLM β*) in total *FLM* transcripts decreased by about 20% upon transfer from darkness to blue light in the WT at 22 °C, and decreased by more than 40% upon transfer from darkness to blue light in the WT at 16 °C; these differences disappeared in the *cry1 cry2* and *cis1-1* mutants, suggesting that blue light represses the accumulation of *FLM β* through CRYs and CIS1 (Fig. 4b). RT-qPCR showed that blue light repressed the splicing efficiency (SE) of *FLM* exon 2 while enhanced the SE of *FLM* exon 3, as transcripts with *FLM* exon 2 were more abundant in 10-day-old seedlings grown at 22 °C LD and 16 °C LD in white light without blue light than in white light containing blue light (Fig. S6i). Cutoff filters were used to filter out all blue light (400–500 nm). RT-PCR or RT-qPCR assays also revealed a CRY-dependent decrease of *FLM* exon 2 abundance in *CIS1* overexpression lines, while transcripts with *FLM* exon 2 were more abundant in *cry2-1*, *cry1 cry2*, *cis1-1*, *cis2-1*, and *cis1 cis2* mutants relative to the WT in 10-day-old seedlings grown at 22 °C or 16 °C LD condition (Fig. 4c and Supplementary Fig. 6j, k). Sanger sequencing of individual *FLM* cDNA clones from 10-day-old seedlings grown at 22 °C LD condition confirmed that overexpression of *CIS1* led to an accumulation of *FLM δ* (~42% of the total, compared to 15% in WT) at the expense of both *FLM β* (~39% compared to 61% in WT) and nonsense-mediated mRNA decay (NMD)-target transcripts (~19% compared to 25% in WT) (Supplementary Data 5). *FLM* was excluded from various sets of DEGs between WT and *cry1 cry2* or between WT and *cis1-1* in both dark and blue-light conditions in our RNA sequencing data (Supplementary Data 1). Relative total *FLM* transcript levels were comparable in WT, *cry1 cry2*, and *cis1-1* seedlings grown in both dark and blue-light conditions, as determined by RT-qPCR (Supplementary Fig. 6l). NMD of *FLM* variants plays an important role in response to thermosensory flowering^25^. However, we established that CRYs and CIS1 have little effect on the decay of *FLM* mRNAs, as *FLM* exon 2 or *FLM* exon 3 products showed similar NMD sensitivity in *cry1 cry2* and *cis1-1* mutants as Col-0 after treatment with cycloheximide (CHX, an inhibitor of translation and of NMD) (Supplementary Fig. 6m). These findings suggested that the CIS1–CRY2 complex regulates *FLM* splicing and inhibits the accumulation of *FLM β* transcripts. Additional CIS1 targets other than *FLM* might also contribute to the observed flowering time differences between WT and *cis1 cis2* mutants. Genes involved in flower development were enriched in GO analysis of differentially spliced genes (Fig. 3d and Supplementary Data 3); *CIRCADIAN CLOCK ASSOCIATED1 (CCA1)*, the circadian clock-related factor was also regulated by blue light and CIS1 (Supplementary Data 4).

### CRY2 and CIS1 regulate flowering via FLM

**Fig. 5:**
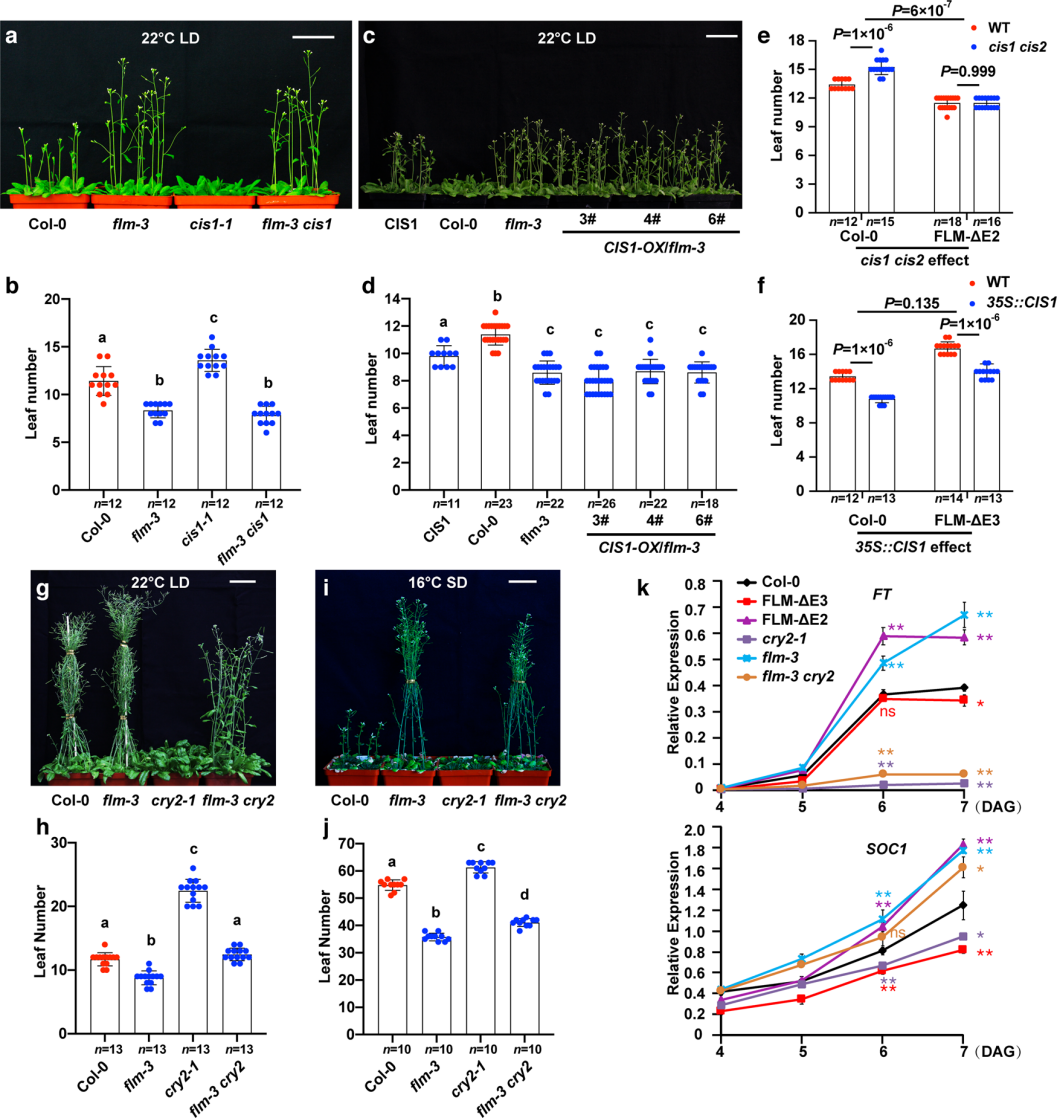
CRY2 and CIS1 regulate thermosensory flowering via FLM. **a**, **c** Representative photographs of 24-day-old (**a**) and 20-day-old (**c**) plants of the indicated genotypes grown at 22 °C in LD conditions. Scale bar = 5 cm. **b** and **d** Number of rosette leaves of the indicated genotypes shown in **a** (for **b**) and **c** (for **d**). Blue and red circles indicate the data from individual plants. Error bars represent the s.d. in (**b**) and (**d**). Lowercase letters indicate statistically significant differences, as determined by one-way ANOVA with Tukey’s multiple comparisons test (*P* < 0.05). **e**, **f** Flowering phenotype of the indicated genotypes grown at 22 °C in LD conditions, reported as the number of rosette leaves at the time of flowering. Blue and red circles indicate the data from individual plants. Error bars represent the s.d., two-sided Student’s *t*-test was used to calculate *P* values. **g**, **i** Representative photographs of 38-day-old plants grown at 22 °C in LD conditions (**g**) and 93-day-old plants grown at 16 °C in SD conditions (**i**). Scale bar = 5 cm. **h** and **j** Number of rosette leaves of the indicated genotypes shown in **g** (for **h**) and **i** (for **j**). Blue and red circles indicate the data from individual plants. Error bars represent the s.d. Lowercase letters indicate statistically significant differences, as determined by one-way ANOVA with Tukey’s multiple comparisons test (*P* < 0.05). **k** RT-qPCR results showing the expression of *FT* and *SOC1* in the indicated genotypes. Seedlings grown at 22 °C in LD conditions were collected at zeitgeber 16 (ZT16, 16 h after lights on) for the time course after germination. Error bars, s.d. of three biological replicates. The asterisks indicate a significant difference from Col-0 based on a two-sided Student’s *t*-test (*P < 0.05, **P < 0.01).

To further explore the relationship between CIS1 and FLM, we investigated the genetic interactions between *CIS1* and *FLM*. We crossed the loss-of-function mutant *flm-3* to the *cis1-1* mutant to generate the *flm-3 cis1* double mutant (Supplementary Fig. 7a). The late flowering phenotype of *cis1-1* was lost in the *flm-3 cis1* mutant background at 22 or 16 °C in LD conditions (Fig. 5a, b and Supplementary Fig. 7b), indicating that FLM acts downstream of CIS1. Overexpression of *YFP-CIS1* in the *flm-3* background did not further accelerate the early flowering phenotype seen in the *flm-3* mutant (Fig. 5c, d and Supplementary Fig. 7c), suggesting that FLM is one of the main CIS1 targets with respect to flowering time control.

In *cis1-1* mutants, *FLM β* transcripts accumulated to higher levels, leading to late flowering; conversely, the overexpression of *CIS1* resulted in both a reduction of *FLM β* transcript abundance and an increase of *FLM δ* transcripts, causing early flowering. To dissect whether CIS1 effects are mediated via *FLM β* and/or *FLM δ*, we carried out a genetic analysis with genome-edited lines lacking either exon 2 (*FLM-ΔE2*) or exon 3 (*FLM-ΔE3*) (Supplementary Fig. 7d–g). The late flowering phenotype of the *cis1 cis2* double mutant was abolished in the *FLM-ΔE2* background (Fig. 5e and Supplementary Fig. 7f, G × G *P* < 0.0001), confirming that the late flowering seen in *cis1 cis2* requires functional *FLM β*. By contrast, we observed a comparable early flowering phenotype associated with the overexpression of *CIS1* in the WT and in the *FLM-ΔE3* background (Fig. 5f and Supplementary Fig. 7g, G × G *P* > 0.05), indicating that the early flowering phenotype caused by *CIS1* overexpression is independent of *FLM δ*. We concluded that CIS1 effects are mediated mainly via *FLM β*.

Furthermore, the late flowering phenotype of the *cry2* mutant was suppressed in the *flm-3 cry2* double mutant at both 22 and 16 °C in LD conditions, at 22 °C in LD under blue-light conditions (Fig. 5g, h and Supplementary Fig. 7h–k) and at 16 °C in SD conditions (Fig. 5i, j). The exaggerated flowering time delay seen in the *cry2* mutant at low temperatures was largely abrogated in the *flm-3 cry2* double mutant (G × E *P* > 0.05), indicating that this phenotype at 16 °C is mainly due to FLM in the absence of CRY2*. SUPPRESSOR OF OVEREXPRESSION OF CO 1* (*SOC1*) were expressed to similar levels in *FLM-ΔE3* and the WT, but was higher in the *flm-3, FLM-ΔE2*, and *flm-3 cry2* backgrounds compared to the WT and the *cry2* mutant, indicating that the higher *SOC1* transcript levels in the *flm-3* mutant are caused by a reduction in *FLM β* levels*. FT* expression was the same in the *flm-3 cry2* and *cry2* mutants (Fig. 5k). These results indicated that FLM acts downstream of CRY2 to regulate flowering.

### CRYs regulates the RNA-binding activity of CIS1 to modulate *FLM* splicing

**Fig. 6:**
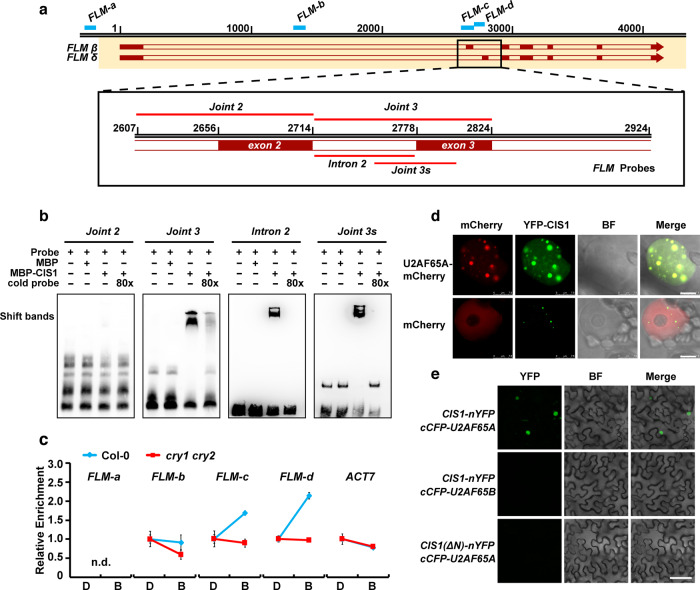
CRYs regulates the RNA-binding activity of CIS1 to modulate *FLM* splicing. **a** Diagram illustrating the locations of RNA probes (red lines) and primer pairs (blue bars) used in RNA-EMSA and RIP assays. **b** RNA-EMSA assay showing that CIS1 binds to the *FLM* Joint 3 probe but not *FLM* Joint 2 in vitro and that CIS1 shows the strongest binding affinity to short *FLM* Joint 3 (*FLM* Joint 3 s) among the short probes (Intron 2 and *FLM* Joint 3 s). RNA probes are indicated in (**a**). **c** RIP-qPCR assay showing the binding affinity of CIS1 protein to *FLM* pre-mRNA in vivo. Col-0, *cry1 cry2*, and *cis1-1* (mock) seedlings were grown at 22 °C in LD condition for 7 days and pretreated with darkness (D) for 24 h, treated with MG132 for 2 h, and transferred to blue light (B, 30 μmol m^−2^ s^−1^) for 4 h. RNA fragments (200–400 nt) extracted from seedlings were immunoprecipitated with anti-CIS1 agarose beads (IP). The precipitated RNA was analyzed by RT-qPCR using different primer pairs of *FLM* pre-mRNA as indicated in (**a**). The *FLM* promoter and *ACT7* served as negative controls. The level of binding was calculated as the ratio between IP and mock, normalized to that of *IPP2* as an internal control; n.d., not detected. Error bars, s.d. of three biological replicates. **d** Co-localization of CIS1 and U2AF65A proteins in nuclear speckles in *N. benthamiana*. mCherry served as a negative control. Scale bar = 5 μm. **e** BiFC assay showing in vivo protein interactions between CIS1 and U2AF65A. *CIS1(ΔN)-nYFP* and *cCFP-U2AF65B* were used as negative controls. *N. benthamiana* leaf epidermal cells were co-infiltrated with the indicated constructs. Scale bar = 20 μm. In **b**, **d**, **e**, three independent experiments were performed with similar results.

To test whether CIS1 physically binds the *FLM* pre-mRNA to regulate its AS, we performed RNA electrophoretic mobility shift assays (REMSAs). CIS1 interacted in vitro with the highest affinity to the joint region between intron 2 and exon 3 of *FLM* pre-mRNA (Fig. 6a, b) but did not bind to the joint region between intron 1 and exon 2. We also conducted in vivo RNA immunoprecipitation assays (RIP) to test the association between CIS1 and the *FLM* pre-mRNA in plant cells. We grew Col-0 and *cry1 cry2* mutant seedlings in dark or blue-light conditions before isolating the nuclear RNA species associated with CIS1 for RT-qPCR quantification of various regions of the *FLM* transcript, using *ACTIN 7* (*ACT7*) as a control transcript. One of the key alternatively spliced regions of *FLM* (exon 2–intron 2–exon 3 regions) was enriched in the immunoprecipitated fraction, in a CRY2-dependent manner and specifically in blue-light conditions (Fig. 6a, c). CIS1 efficiently co-immunoprecipitated *FLM-c* (intron 1/exon 2 junction region) and *FLM-d* (intron 2–exon 3 junction region) but not *FLM-a* (the promoter region of *FLM*), *FLM-b* (intron 1 of *FLM*), or *ACT7* (Fig. 6a, c). We concluded that blue light promotes the association of CIS1 with *FLM-c* and *FLM-d* in a CRY-dependent manner, since the RNA-binding activity of CIS1 was higher in blue light than in darkness, while we observed no difference between darkness and blue-light conditions in the *cry1 cry2* double mutant (Fig. 6c). These results indicated that CIS1 associates with the *FLM* pre-mRNA in vivo to regulate AS of *FLM* transcripts in blue light- and CRY-dependent manner.

To test the contribution of CRY2 to CIS1-*FLM* binding at different temperatures, we conducted in vivo RNA immunoprecipitation assays using transgenic seedlings expressing the constitutively active variant CRY2^W347A^ in the *cry1 cry2* mutant background (CRY2^W347A^/*cry1 cry2*) or the flavin-deficient variant CRY2^D387A^ (CRY2^D387A^/*cry1 cry2*). We grew seedlings for 7 days at 16 °C in continuous blue light before transferring them to 22 °C for 10 h and harvesting tissue for CIS1 immunoprecipitation. RT-qPCR results detected more *FLM-c* and *FLM-d* enriched among the immunoprecipitated fraction from CRY2^W347A^ */cry1 cry2* at 22 °C than at 16 °C, but comparable amounts of the two isoforms *FLM-c* and *FLM-d* from CIS immunoprecipitates of CRY2^D387A^/*cry1 cry2* at 22 and 16 °C (Supplementary Fig. 8a). These results indicated that blue light and higher temperatures enhance CIS1-*FLM* accumulation in vivo and that the ability of CIS1 to bind to *FLM* is regulated by light-activated CRY2.

Mammalian Splicing Factor 1 (SF1) is required for pre-spliceosome assembly. SF1 interacts with the SURP domain via a conserved domain in SF1, and the SF1-SURP interaction is required for efficient early spliceosome assembly^34^. Since CIS1 harbors a SURP domain, we tested whether CIS1 interacted with the Arabidopsis SF1 homolog (At5g51300). CIS1 interacted with Arabidopsis SF1 in BiFC assays (Supplementary Fig. 8b). CIS1 also interacted with U2AF65A, the U2-associated splicing factor that recruits the U2 snRNP to the spliceosome CIS1 co-localized with U2AF65A in nuclear speckles in *N. benthamiana* leaves (Fig. 6d). Furthermore, CIS1 interacted with U2AF65A in *N. benthamiana* cells, as revealed by BiFC assays (Fig. 6e). CIS1 did not interact with U2AF65B, while CIS1(△N) (lacking the CIS1 N terminus) did not interact with U2AF65A by BiFC assay (Fig. 6e), and thus served as negative controls for the BiFC assays. These results indicate that CIS1 associates with U2 components and may be involved in the assembly of the pre-spliceosome on a certain group of pre-mRNAs to regulate AS.

**Fig. 7:**
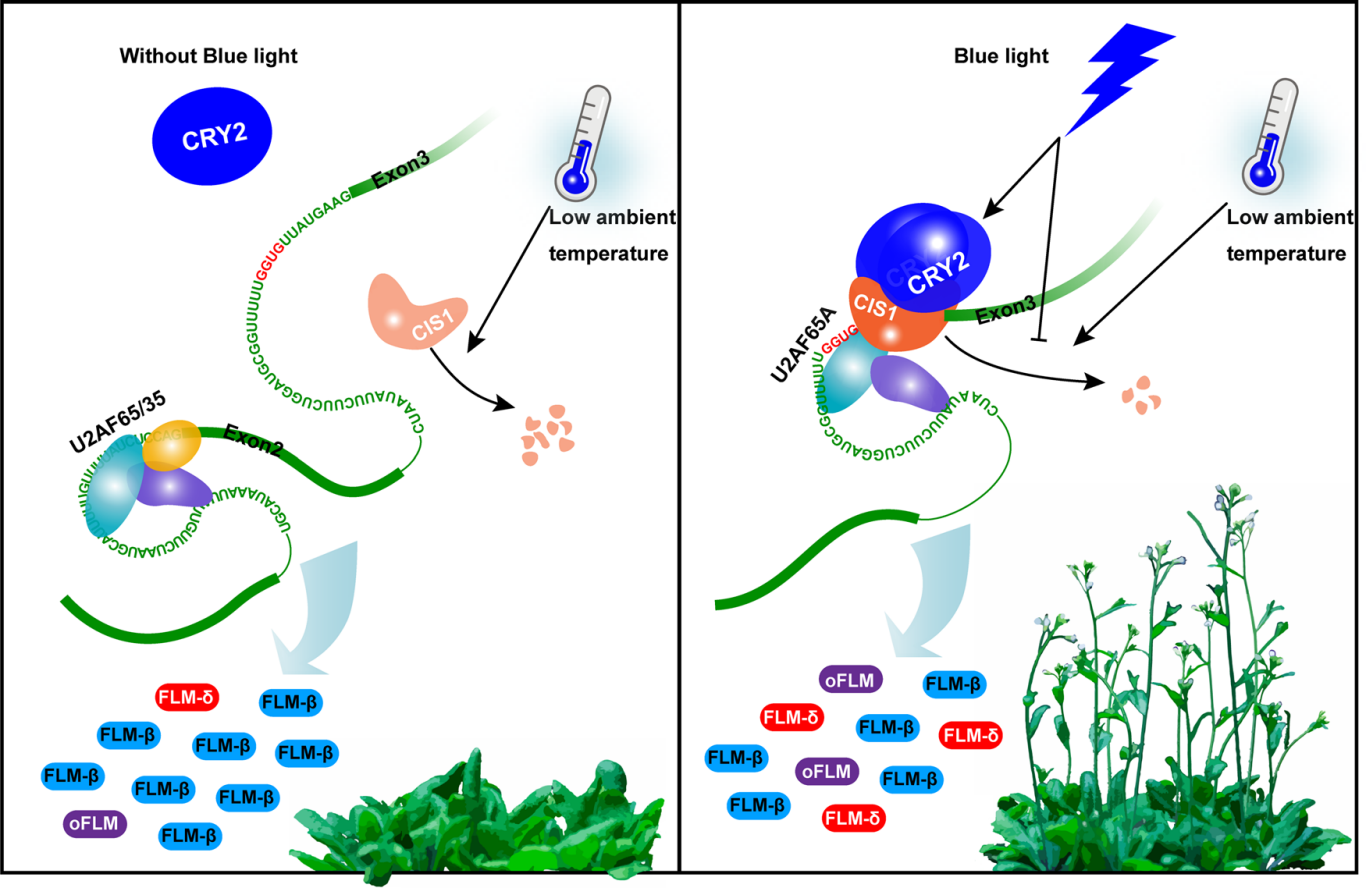
A hypothetical model for blue-light regulation of thermosensory flowering via the CRY2–CIS1–FLM signaling pathway. At low ambient temperatures without blue light, CRY2 is inactive, CIS1 protein is unstable, the 3ʹ splice site of the second *FLM* intron is a weak AS site because of the G tract sequence, leading to more accumulation of *FLM β*. When there is blue light, CIS1 protein is stabilized; photoexcited CRY2 forms homodimers and interacts with CIS1 to promote its binding to intron 2 or exon 3 in the *FLM* pre-RNA. CIS1 also interacts with SF1 and U2AF65A to recruit U2-snRNP to promote the accumulation of non-*FLM β* including *FLM δ* and other *FLM* variants (*oFLM*).

The polypyrimidine^35^ tract is close to the 3ʹ AG that binds the U2AF65 and U2AF35 heterodimer at the 3ʹ splice sites at the end of introns^36^. There was a G tract near the Py tract of the *FLM* second intron that was reported to be a splicing silencer enriched in genes involved in cancer and highly associated with AS in mammals^37^ (Supplementary Fig. 8c). The working model is that without CIS1, the 3ʹ splice site of the second intron behaved as a weak AS site because of the G tract, resulting in greater accumulation of *FLM β*; when present, CIS1 interacted with SF1 and U2AF65A and *FLM* intron 2 to recruit the U2 snRNP and promote the accumulation of non-*FLM β* transcripts, including *FLM δ* (Fig. 7). Photoexcited CRY2 modulated the RNA-binding activity of CIS1, whereas the protein stability of CIS1 was regulated by both ambient temperature and blue light.

## Discussion

CRY2 primarily mediates photoperiodic regulation of floral initiation, as *cry2* mutants show a very late flowering phenotype in LD but not SD conditions when grown at 22 °C^2^. CRY2 mediates photoperiodic control of floral initiation in response to blue light by at least three distinct mechanisms: suppression of CO degradation by associating with the COP1 complex^12,13,15–17^; direct activation of CIB transcription factors^8–10,38^; and regulation of light entrainment of the circadian clock^39^. Here we show that CRY2 is involved not only in photoperiodic flowering but also in thermosensory flowering, as *cry2* mutants flower normally at 22 °C in SD conditions but showed delayed flowering at 16 °C in SD conditions. The CRY2–CIS1–FLM module identified here is a previously unrecognized flowering control pathway that allows light and ambient temperature signaling to coordinately regulate flowering. Blue light and CRYs are required to repress hypocotyl elongation promoted by high ambient temperatures^40^, for temperature compensation of the circadian clock^41^, for temperature-dependent internode elongation^42^, and for maintenance of plant biomass^43^. CRY2 protein abundance is modulated by ambient temperature, with CRY2 being degraded at low ambient temperatures via the 26S proteasome^7^. Here we show that (1) blue light and CRYs promote flowering even in SD conditions at low ambient temperatures at least partially by preventing the accumulation of *FLM β* transcripts; (2) CIS1 protein stability is regulated by both ambient temperature and blue light; and (3) the RNA-binding activity of CIS1 toward *FLM* is modulated by CRYs. Other CIS1 targets in addition to *FLM* might contribute to the flowering time difference between WT and the *cis1 cis2* mutant.

CRYs are present in all three major evolutionary lineages (bacteria, plants, and animals), although their roles as photoreceptors remain controversial in mammals^44–46^. CRY2 interacts with the bHLH transcription factors CIB1 and PHYTOCHROME-INTERACTING FACTOR 4 (PIF4) to regulate transcription^8,9,38,40,47^. Similarly, mammalian CRYs interact with the bHLH proteins CLOCK/BMAL to suppress their activation of E box–dependent transcription^48^. The interaction between CRYs, bHLH proteins, and the G-box motif appears to be conserved from plants to animals. Arabidopsis CRYs also associate with the COP1 complex to modulate protein stability^12,13,15–17^. Whether or how CRYs might affect other steps besides transcription and protein stability is a major challenge in our understanding of CRY-mediated photoresponses^18^. Here we show that CRY2 physically interacts with the splicing factor CIS1 in a blue light-dependent manner to regulate RNA AS. Pre-mRNA splicing is a key step in the posttranscriptional regulation of transcript levels. AS greatly expands the functional proteome repertoire of eukaryotic organisms^19^. Our results indicate that environmental light signals and photoactivated blue-light receptor CRYs regulate gene expression via multiple mechanisms: transcriptional regulation, protein stability, and also AS. Phytochromes are involved in regulating pre-mRNA splicing^49^ and significantly modulate global AS profiles in different species^22,50^. SFPS, a protein related to human splicing factor 45 (SPF45), mediates phytochrome-regulated seedling development by modulating pre-mRNA splicing of light signaling and circadian clock genes^21^. REDUCED RED LIGHT RESPONSES IN *cry1 cry2* BACKGROUND 1 (RRC1), an orthologous protein to the potential human splicing factor SR140, is also involved in phyB-mediated AS of light-responsive genes^51^. In the moss *Physcomitrium* (*Physcomitrella*) *patens*, phytochrome 4 physically interacts with splicing regulators including heterogeneous nuclear ribonucleoprotein H1 (PphnRNP-H1) or F1 (PphnRNP-F1) in the nucleus to regulate light-responsive AS^50,52^ The interaction between photoreceptors and splicing factors thus appears to have evolved early to provide light-regulated AS for optimum photomorphogenesis and light-regulated development.

CIS1 is the first splicing factor that interacts with CRYs in a blue light-dependent manner. *CIS1* transcription is regulated by blue light. CIS1 protein abundance is also modulated by both blue light and temperature, the same as CIS1, CRY2 protein accumulates at high ambient temperature and gets degraded at low ambient temperatures via the 26S proteasome^7^, indicating that there might be more CRY2–CIS1 protein complex at high ambient temperature than at low ambient temperature. Furthermore, photoactivated CRY2 affects its RNA-binding activity. How CRY2 regulates CIS1 binding to FLM transcript is not quite clear. Our results show CIS1 interacts with U2AF65A and SF1 but not U2AF65B (Fig. 6d, e and Supplementary Fig. 8b). It had been reported that the loss-of-function mutant of U2AF65A^53^ or SF1^54^ exhibit AS of FLM and flowering phenotype. In *atsf1-2* mutants, *FLM β* decreased significantly but *FLM δ* marginally increased. In *atu2af65a-4* mutants, *FLM β* significantly increased but *FLM δ* not altered. CRY2–CIS1 was added as an indispensable puzzle of the molecular mechanism of FLM AS. CIS1 integrates environmental blue light and ambient temperature signals to modulate AS. CIS1 is highly conserved during evolution (Supplementary Fig. 2a), as are CRYs. Our results indicate that human CRY2 interacts with 4 human CIS1 homologs among the 14 hCIS1 tested, including the putative splicing factor SUGP1, reported to be involved in the regulation of cholesterol metabolism^31^ (Supplementary Fig. 2b). We conclude that the interaction between CRYs and G-patch domain-containing RNA-binding proteins may be evolutionarily conserved between plants and mammals for the regulation of pre-spliceosome assembly and pre-mRNA splicing, and therefore to regulate gene expression and proteome diversity. The activated plant CRY-PHR forms an active dimer both in cryo-EM and crystal structures, mutations that influence the conformational changes of CRY dimerization are likely to change the blue light signal transduction process^5^. It is noteworthy that similar conformational changes in plant CRYs have not been found in mammalian^55,56^ or drosophila CRYs^57,58^, consistent with that CRY2 interacts with CIS1 in a blue light-dependent manner while human CRY2 interacts with human CIS1 homologs both in darkness and blue light conditions. G tracts are reported to act as splicing silencers, as this motif was enriched in genes involved in cancer and highly associated with AS in mammals^37^. The second intron of *FLM* contained a G tract near the Py tract, where CIS1 interacted with SF1 and U2AF65A to recruit the U2 snRNP to this weak AS site and promote the accumulation of non-*FLM β* transcripts. Our results provide an example of how a G tract is selected for AS.

In summary, we show here that photoactivated CRYs interact with the splicing factor CIS1 to regulate its RNA-binding activity and transcriptome-wide AS, including that of the floral regulator transcript *FLM*, thus linking light signals to ambient temperature-modulated flowering.

## Methods

### Plant materials and growth conditions

Except where indicated, the Columbia ecotype of *Arabidopsis thaliana* was used. The mutants *cry2-1*, *cry1 cry2* and *flm-3* were previously described^2,27,59^. T-DNA insertion mutants *cis1-1* (SALK_087941C), *cis1-2* (SALK_000942C), and *cis2* (SALK_041197C) were obtained from the Arabidopsis Biological Resource Center (ABRC). The double mutants *cis1-1 cis2-1*, *flm-3 cis1-1*, *flm-3 cry2*, and triple mutants *flm-3 cis1-1 cis2* were prepared by genetic crosses, and their identities were verified by genotyping.

The two edited FLM CRISPR lines FLM-ΔE2 (which carries a deletion of 57 bp that covers most of exon 2) and FLM-ΔE3 (which carries a 64-bp deletion that completely covers exon 3) were constructed by us. The egg-cell-specific promoter-controlled CRISPR/Cas9 system^60^ was used, and the sgRNA designed by Capovilla, G. et al.^61^ was cloned into pHEE401-2gR. The construct was transformed into plants via the floral dipping method^62^. Plants carrying the deletion were identified by PCR and Sanger sequencing.

The full-length *CIS1* coding sequences were cloned into pEarly-104 (ABRC) or pEGAD-Myc vector^8^, bearing either *YFP* (*Pro35S::YFP-CIS1*) or *Myc* (*Pro35S::Myc-CIS1*), and transformed into different genetic backgrounds, including WT (Col-0), the *cry1 cry2* double mutant and *flm-3*. To study the promoter activity of the *CIS1* gene, a 2002-bp genomic DNA fragment of the *CIS1* gene (−2002 to −1) was amplified by PCR, cloned into pTF102 to result in the *CIS1::GUS* construct, and transformed into WT by floral dipping^62^. For functional complementation tests, the full-length *CIS1* coding sequences driven by the *CIS1* promotor (−2002 to −1) were cloned into pEarly-302 (ABRC) and transformed into *cis1-1* mutant plants by floral dipping^62^.

For every transformation, more than ten independent transgenic lines with a single copy of the transgenes were generated. Immunoblots were performed to verify the overexpression of the transgenes. Phenotypes of transgenic plants were verified in at least three independent transgenic lines.

The raw values for the flowering time measurements used in all figures are provided in the Source data.

### RNA-seq and transcriptome analysis

For RNA-seq, WT, *cry1 cry2*, and *cis1-1* seedlings were grown for 4 days in the dark and then exposed to continuous blue light (30 μmol m^−2^ s^−1^) for 3 h; one batch of etiolated seedlings was kept in darkness for the additional 3 h as a dark control. The seedlings were harvested, and total RNA was isolated using RNAiso Plus (Takara). Three biological replicates were independently prepared throughout the processes, from the induction of seed germination to the preparation of mRNA-seq libraries. The RNA library generation process followed the manufacturer’s protocol for the Illumina TruseqTM RNA sample prep Kit. The average RNA fragment was about 300 bp, and a 15-cycle PCR amplification was carried out with the primer mixture provided in the kit. Library preparation and sequencing using an Illumina Hiseq4000 instrument with 2 × 150-bp paired-ends reads were performed by Majorbio (Shanghai). RNA-seq data were mapped with STAR v2.5.2 using minimum intron size 20, and maximum intron size 6000. A splice junction BED file was generated using RegTools v0.5.2 ‘junctions extract’ command with the same intron limits^63^. Each mapped RNA-seq sample was processed with SpliSER v0.1.1 and a separate combine command was called for each comparison^32^. SpliSER output was analyzed for differential splicing using the diffSpliSE pipeline. Differentially Spliced sites were called those with a *p*-value < 0.05 or FDR-corrected *p*-value < 0.05, and an absolute change in averaged SSE > 0.1 between conditions (Supplementary Data 2 and 3). To maintain the accuracy of the quantification, a splice site would be filtered out unless each replicate being assessed had at least 10 reads covering the site.

For differential gene expression analysis, read counts were generated from RNA-seq alignments using featureCounts v1.5.1. Differential gene expression was called using DESeq2 v1.22.2 with read counts normalized using the sizeFactors function^64^ (Supplementary Data 1). Genes with a corrected *p*-value < 0.05 and absolute log2FoldChange > 2 were taken as differentially expressed. Overlaps between gene lists were tested through hypergeometric probabilities.

### Phylogenetic analysis

Amino acid sequences were downloaded from National Center for Biotechnology Information (NCBI) and aligned using MUltiple Sequence Comparison by Log-Expectation (MUSCLE) in the MEGA7 software package with the default settings for protein multiple alignments. Evolutionary distances were computed using Poisson correction analysis. The bootstrap method with 1000 replicates for phylogeny testing was used. The cluster analysis of G-patch domain-containing proteins was conducted in MEGA7 using the Neighbor-Joining method and the bootstrap consensus tree inferred from 2000 replicates. The prediction of domains was analyzed in the NCBI BLAST conserved domains section^65^.

### mRNA expression analyses and GUS staining

Total RNAs were isolated using RNAiso Plus (Takara). cDNA was synthesized from 500 ng total RNA using PrimeScript RT Reagent Kit with gDNA Eraser (Takara). TB Green Premix Ex Taq (Takara) was used for qPCR, on the MX3000 System (Stratagene). The level of *ACTIN7* mRNA (AT5G09810; Supplementary Data 6) was used as the internal control. Primer efficiency was considered when measuring differences in expression and the PCR conditions are listed together with every pair of primers (Supplementary Data 6). The two main *FLM* transcript isoforms were quantified using ImageJ software package. The expression of GUS (β-glucuronidase) was analyzed as described^66^.

### Yeast two-hybrid analysis (Y2H)

The Y2H screening assays were performed according to the manufacturer’s instructions (Matchmaker user’s manual, Clontech, California). The sequences encoding CRY2, CRY2^W374A30^, CRY2^D387A^, CRY2N375 which contain residues 1–375, CRY2N565 (residues 1–565) and CRY2N489 (residues 1–489)^67^ were fused in-frame with that encoding the GAL4 DNA binding domain (BD) of the bait vector pBridge (Clontech). The Arabidopsis cDNA library cloned in the prey vector pACT was obtained from Dr. Joe Ecker (Salk Institute, California). The bait plasmid pBridge-CRY2 and the prey plasmids or library DNA were co-transformed into yeast strain Y190. Approximately 1 × 10^7^ transformants were screened each time to select colonies that grew under blue light on a medium without histidine but with 3-AT (50 mM). The prey plasmid DNAs were isolated from the yeast and transformed into *E. coli*; the plasmids were then isolated from the *E. coli* and re-transformed into yeast cells containing only the bait plasmid to further verify the phenotype. The clones that continued to grow in blue light but not in the dark in the His− and 3AT+ medium after retransformation were selected for DNA sequence analyses.

The full-length *CIS1* cDNA was PCR amplified from Arabidopsis RNA, sequenced, and cloned into pGADT7 (Clontech) for further analyses. The bait plasmids and the prey plasmids were co-transformed into yeast strain Y190. Light-dependent CRY2–CIS1 interaction was quantified using the quantitative β-galactosidase assay; to maintain the cell density between 0.4–0.8 OD_600_ throughout the experiment, yeast cells were grown overnight in −Leu/−Thr medium, diluted 5-fold in YPD medium, grown in the dark for 1–2 h, transferred to blue light or left in the dark as controls, and grown for up to 180 min, during which samples were taken for the β-galactosidase assay. To analyze CRY2 fragments and CIS1 interaction by the β-galactosidase assay, yeast colonies were patched in duplicate onto −Leu/−Thr plates. One duplicate was grown under blue light (30 μmol m^−2^ s^−1^) at 28 °C for 2–3 days. The second duplicate was wrapped in aluminum foil to block the light and grow under the same conditions. Then samples were taken for the β-galactosidase assay.

The sequences encoding CIS1-A which contains residues 1–219, CIS1-B (residues 220–443), CIS1-C (residues 1–431), CIS1-D (residues 1–423), CIS1-E (residues 260–443), CIS1-F (residues 318–443), CIS1-G (residues 356–443) and CIS1-H (residues 380–443) were fused in-frame with those encoding the GAL4 activation domain (AD) of the prey vector pGADT7 (Clontech). The bait plasmids and the prey plasmids were co-transformed into yeast strain AH109. To analyze CRY2^W374A^ and CIS1 fragment interaction via the histidine auxotrophy assay, yeast colonies were patched in duplicate onto −Leu/Thr and −Leu/Thr/His/Ade plates. One duplicate was grown under blue light (30 μmol m^−2^ s^−1^) at 28 °C for 2–3 days. The second duplicate was wrapped in aluminum foil to block the light and grow under the same conditions.

To check the interactions between *Homo sapiens* CRY2 and G-patch domain-containing proteins, the sequences encoding hSUGP1, hSUGP2, hGpatch1, hGpatch2.2, hGpatch2L.1, hGpatch2L.2, hGpatch3, hGpatch4.2, hGpatch5, hGpatch6.1, hGpatch6.4, hGpatch6.5, hGpatch7, hGpatch8.1, hGpatch9.1, hGpatch10, hGpatch11.1 and hGpatch11.2 were cloned into pGADT7. The sequence encoding hCRY2 was cloned into pBridge. The bait plasmids and the prey plasmids were co-transformed into the yeast strain AH109. To analyze their interactions via the histidine auxotrophy assay, yeast colonies were patched in duplicate onto −Leu/−Thr and −Leu/−Thr/−His/−Ade plates and grown at 28 °C for 2–3 days.

### BiFC and co-localization assays

The BiFC assay was based on that described previously with slight modifications^40,68^, constructs for expression of CIS1 or CRY2, U2AF65A, SF1 fused to the C-terminus of CFP(155-238) or N-terminus of YFP(1–172) were transformed into *Agrobacterium* strain GV3101. For co-localization, constructs for expression of CRY2 or U2AF65A fused to mCherry, and CIS1 fused to YFP were transformed to *Agrobacterium* strain GV3101. Overnight cultures of agrobacteria were collected by centrifugation, resuspended in MES buffer to 0.6 OD_600_, mixed with GV3101 expressing pSoup-P19, and incubated at room temperature for 2 h before infiltration. Agrobacteria suspensions in a 1-mL syringe (without the metal needle) were carefully press-infiltrated manually onto healthy leaves of 3-week-old *Nicotiana benthamiana*. Plants were left under long days (LD, 16-h light/8-h dark) and white light conditions for 3 days after infiltration.

### BiLC assays

*CRY2* or *CIS1* and *CIS1* paralogue genes *CIS2* (At1g63980), At1g17070, At2g42330, At3g52350, At5g08535, At1g33520, At4g25020, At3g09850, At3g57910, At1g30480, At3g54230, At4g34140, At2g24830, and At5g26610 were fused to sequences encoding the C- or N-terminus of firefly luciferase and transformed into *Agrobacterium* strain GV3101. *Nicotiana benthamiana* plants were left under LD white light conditions for 3 days after infiltration. The leaves were infiltrated with luciferin solution (1 mM luciferin and 0.01% Triton X-100), and images were captured using a CCD camera 5 min later.

### Immunoblot and co-immunoprecipitation (co-IP)

Immunoblotting was carried out as described previously^69^. Anti-CIS1 antibody is a custom-made antibody, the coding sequence of *CIS1* was cloned into pET28a (Novagen). Protein expression, purification, and antibody production were performed by ABclonal (Wuhan). The anti-CIS1 (ABclonal, #WG-02480D E7293) antibody was used at 1:2000; the anti-Myc (Millipore, #05-724) antibody or anti-GFP (Abicode, #M0802-3a) antibody was used at 1:3000.

For co-IP, 6-day-old Col-0, *cry2*, and *cis1-1* seedlings grown under long-day conditions were pretreated in dark for 24 h, then were treated with 50 μM MG132 for 4 h in the dark followed by exposure to continuous blue light (30 μmol m^−2^ s^−1^) for 20 min; one batch of Col-0 seedlings was kept in darkness for the additional 20 min as a dark control. These samples were harvested, and ground in liquid nitrogen, homogenized in extraction buffer [50 mM Tris (pH 7.6), 150 mM NaCl, 5 mM MgCl_2_, 10% Glycerol, 0.1% NP40, 5 mM DTT, 1 mM PMSF and protease inhibitor cocktail tablets], incubated at 4 °C for 5 min, run through a 1-ml syringe twice (with a metal needle) to promote nuclear lysis, and centrifuged at 14,000 × *g* for 10 min. The supernatant was mixed with 2.5 μL of anti-CIS1 antibody, incubated at 4 °C for 15 min, then mixed with 35 μL protein-A Sepharose (GE), incubated at 4 °C for another 15 min, and washed twice with extraction buffer. The bound proteins were eluted from the beads with 4 × SDS/PAGE sample buffer and analyzed by immunoblot.

### RNA electrophoretic mobility shift assays (REMSAs)

For probes, the RNA transcripts were produced by in vitro transcription using *T7* Promotor drove DNA fragments corresponding to the RNA of interest with the TranscriptAid™ T7 High Yield Transcription Kit (Thermo Scientific), with a modified molar ratio of Bio-11-UTP to standard UTP of 1:12. The probes were heat treated at 85 °C for 8 min, followed by rapid cooling on ice immediately prior to the binding reaction. For protein, the coding sequence of *CIS1* was cloned to pMAL-c5X vector (NEB), expressed, and purified with Amylose Resin (NEB). The binding reaction was carried out in 20 μL binding buffer [10 mM HEPES (7.3), 20 mM KCl, 1 mM MgCl_2_, 1 mM DTT, 5% glycerol, 0.1 mg/ml BSA, 2 μg tRNA] with 25 ng biotin-labeled RNA, 2 μg unlabeled RNA and 500 ng protein were added as indicated. After 20 min incubation at room temperature, the reactions were resolved by 6% native polyacrylamide gel at 4 °C. The binding reaction and detection of the biotin-labeled RNA were carried out using the LightShift Chemiluminescent RNA EMSA Kit (Thermo Scientific).

### RNA immunoprecipitation assays

RIP experiments were modified from ChIP experiments described previously with minor modified^8^. 7-day-old Col-0, *cry1 cry2*, and *cis1-1* (as mock) seedlings grown under 22 °C LD conditions were pretreated in dark for 24 h, then were treated with 50 μM MG132 for 2 h in the dark followed by exposure to continuous blue light (30 μmol m^−2^ s^−1^) for 4 h; one batch of these seedlings was kept in darkness for the additional 4 h as a dark control. Starting material (1 g) was harvested and cross-linked with 1% formaldehyde (Sigma) for 10 min under a vacuum. Cross-linking was stopped by the addition of glycine to a final concentration of 0.125 M. The seedlings were rinsed with water, frozen in liquid nitrogen, and ground to a fine powder. Then the powder was homogenized in Nuclear Extraction Buffer 1 [10 mM Tris–HCl (pH 8.0), 0.4 M sucrose, 10 mM MgCl_2_, 0.1 mM PMSF, cocktail, and 40 U/mL RNase inhibitor]. Nuclei were precipitated by centrifugation at 2000 g for 20 min, washed with nuclear extraction buffer 2 [10 mM Tris–HCl (pH 8.0), 0.25 M Sucrose, 10 mM MgCl_2_, 1% Triton X-100, 0.1 mM PMSF, cocktail and 40 U/mL RNase inhibitor], and lysed in nuclei lysis buffer [50 mM Tris–HCl (pH 8.0), 10 mM EDTA, 1% SDS, 0.1 mM PMSF, cocktail and 160 U/mL RNase inhibitor]. RNAs were sonicated with 7 × 10 s on and 20 s off at low amplitude on the Branson sonifier, sheared to approximately 200–400 bp. The RNA solution was diluted 10-fold with ChIP Dilution Buffer [16.7 mM Tris–HCl (pH 8.0), 167 mM NaCl, 1.1% Triton X-100, 1.2 mM EDTA, 0.1 mM PMSF, cocktail and 200 U/mL RNase inhibitor] and then pre-cleared on a rotator with 20 μL Protein A agarose beads to remove nonspecific associations. Anti-CIS1 antibody was mixed with the RNA solution and incubated at 4 °C overnight, then mixed with prewashed 30 μL Protein A agarose for 2 h. Immunocomplexes were precipitated and washed four times with Binding/Washing Buffer [20 mM Tris–HCl (pH 8.0), 150 mM NaCl, 0.1% SDS, 1% Triton X-100, 2 mM EDTA, 0.1 mM PMSF and 40 U/mL RNase inhibitor]. The bound RNA fragments were eluted with RIP Elution Buffer [100 mM Tris–HCl pH 8.0, 10 mM EDTA, 1% SDS, and 100 U/mL RNase inhibitor] with 50 μg Proteinase-K (Thermo) for each sample, incubated 0.5 h at 55 °C. The RNA was purified with an equal volume of acidic phenol/chloroform/isoamyl alcohol (25:24:1, pH 4.5) and precipitated with 2 vol of ethanol 100% at –70 °C overnight. The samples were centrifuged at 16,000 rpm for 20 min at 4 °C to recover the RNA and resuspended in 10 μL Rnase-free water. The gDNA Erase treatment and RT-PCR were carried out according to the RT reagent Kit with gDNA Eraser (Takara) using 5 μL RNA samples and using Random Hexamer primer (0.2 μg each reaction). qPCRs were performed using 0.5 μL cDNA as a template). The primer pairs used in the RIP experiments are listed in Supplementary Data 6.

### Accession numbers

Sequence data from this work can be found in the Arabidopsis Information Resource or GenBank databases under the following accession numbers: AT3G52120 (CIS1), AT1G63980 (CIS2), AT1G17070 (STIPL1), AT1G30480, AT1G33520 (MOS2), AT2G24830, AT2G42330, AT3G09850, AT3G52350, AT3G54230, AT3G57910, AT4G25020, AT4G34140, AT5G08535, AT5G26610, AT5G51300 (SF1), AT4G36690 (U2AF65A), and AT1G60900 (U2AF65B); genes from *Homo sapiens* can be found in NCBI under the following accession numbers: CRY2, SUGP1, SUGP2, Gpatch1, Gpatch2, Gpatch2L, Gpatch3, Gpatch4, Gpatch5, Gpatch6, Gpatch7, Gpatch8, Gpatch9, Gpatch10, and Gpatch11. RNA-seq data are available from National Center for Biotechnology Information Gene Expression Omnibus (http://www.ncbi.nlm.nih.gov/geo) under the series entries GSE196648.

### Statistical analysis

For flowering phenotype analysis, gene-expression analysis, and RT-qPCR validation of Diff_SpliSE in RNA-seq data, statistical analysis was assessed as described in the figure legends. *P* values were calculated by two-sided Student’s *t*-tests or by one-way ANOVA with Tukey’s multiple comparisons tests using GraphPad Prism9 and were shown in bar graphs or source data. Statistical analysis of RNA-seq data was described in the “Methods” section “RNA-seq and transcriptome analysis”.

### Reporting summary

Further information on research design is available in the Nature Portfolio Reporting Summary linked to this article.

## Supplementary information

Supplementary Information

Description of Additional Supplementary Files

Supplementary Data 1

Supplementary Data 2

Supplementary Data 3

Supplementary Data 4

Supplementary Data 5

Supplementary Data 6

Reporting Summary

## Data Availability

All data needed to evaluate the conclusions in the paper are present in the paper and/or the Supplementary Materials. RNA-seq data are available from National Center for Biotechnology Information Gene Expression Omnibus under the series entry GSE196648. Source data are provided with this paper.

## Source data

Source Data
