# Supplementary material for: CRY2 interacts with CIS1 to regulate thermosensory flowering via FLM alternative splicing
Source: Nat Commun. 2022 Nov 17;13:7045. doi: 10.1038/s41467-022-34886-2 (PMC9671898; doi:10.1038/s41467-022-34886-2)
Supplement: Supplementary file 1 — Supplementary Information [file 41467_2022_34886_MOESM1_ESM.pdf]

Supplementary Materials for

CRY2 interacts with CIS1 to regulate alternative splicing

Zhiwei Zhao, Craig Dent, Huafeng Liang, Junqing Lv, Guandong Shang, Yawen Liu, Fan Feng, Fei Wang, Junhong Pang, Xu Li, Libang Ma, Bing Li, Sridevi Sureshkumar, Jia-Wei Wang, Sureshkumar Balasubramanian and Hongtao Liu*
*Corresponding author. Email: htliu@cemps.ac.cn

This PDF file includes:

Supplementary Fig. 1 to 8

Other Supplementary Materials for this manuscript include the following:

Supplementary Data 1 to 6 (.xlsx)
Supplementary Data 1. List of differentially expressed genes
Supplementary Data 2. List of differentially spliced genes
Supplementary Data 3. List of GO terms in the differentially spliced genes
Supplementary Data 4. Genes showing AS in a blue light– and CIS1–dependent manner
Supplementary Data 5. Different splice forms identified through Sanger sequencing
Supplementary Data 6. Primers list

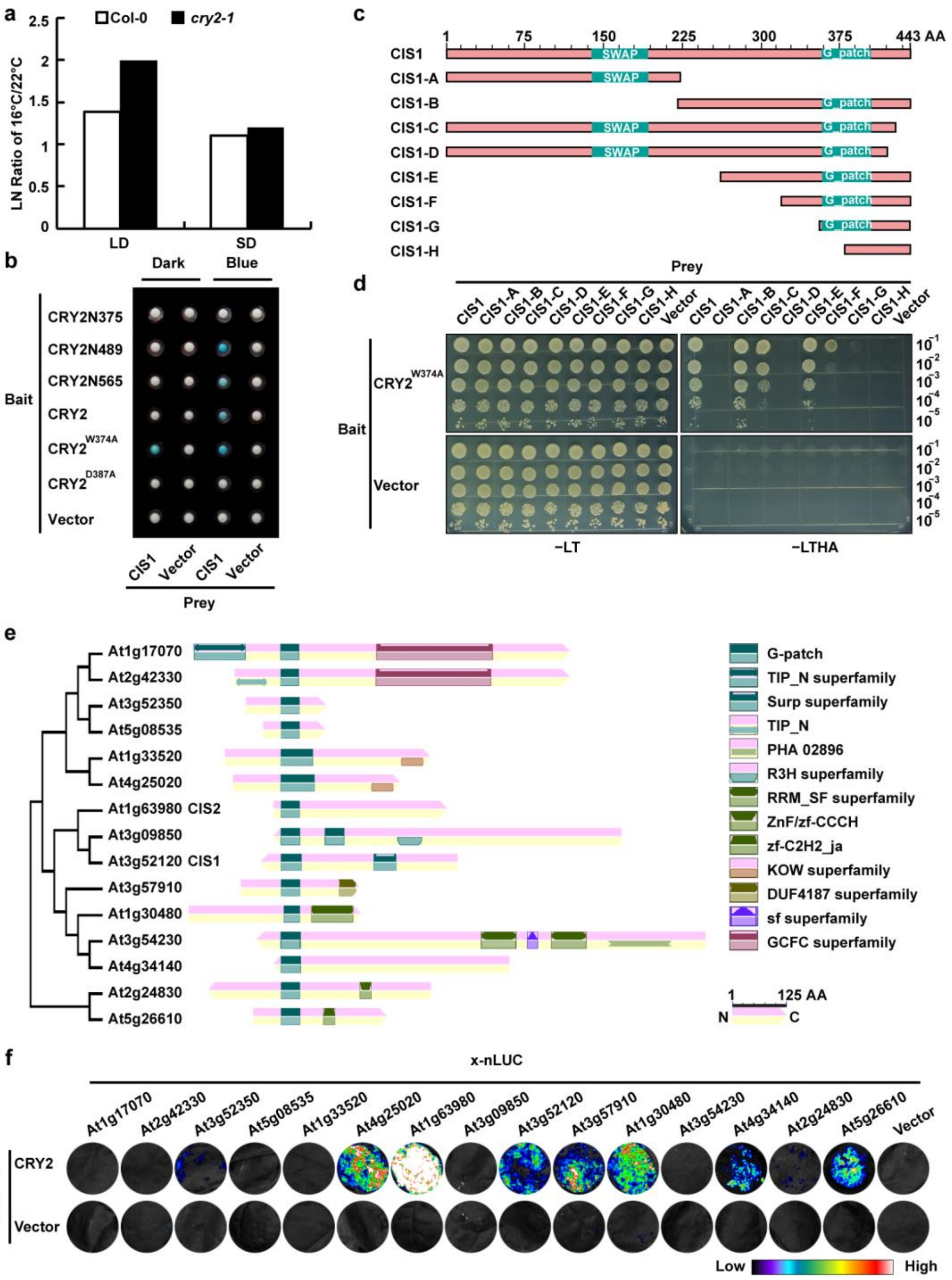

Fig. S1. Blue light-dependent interaction between CRY2N489 or N565 and CIS1C, and multiple G-patch-containing proteins interact with CRY2 in Arabidopsis.

(**a**), Leaf number ratio of 16°C/22°C for Col-0 and *cry2-1* at LD and SD conditions. (**b**), The β-gal assay showing blue light-dependent interaction between CRY2N489 (1-489 aa) or CRY2N565 (1-565 aa) and CIS1, and the lack of interaction between CRY2N375 (1-375 aa) and CIS1. $CRY2^{W374A}$ is a constitutively active site-specific mutant of CRY2. $CRY2^{D387A}$ is a site-specific mutant of CRY2 that cannot be activated by blue light. Yeast cells were grown at –LT medium in the dark or under blue (blue light 30 $\mu mol \cdot m^{-2} \cdot s^{-1}$). (**c**), Diagram depicting the CIS1 fragments

used in **d**. (**d**), Histidine auxotrophy assays showing the interaction between CIS1C260-443aa and CRY2^{W374A}. (**e**), Cluster analysis of G-patch domain-containing proteins in Arabidopsis. (**f**), BiLC assay showing that CRY2 interacts with multiple G-patch-containing proteins. Leaf epidermal cells of *Nicotiana benthamiana* were co-transformed with fusion proteins as indicated.

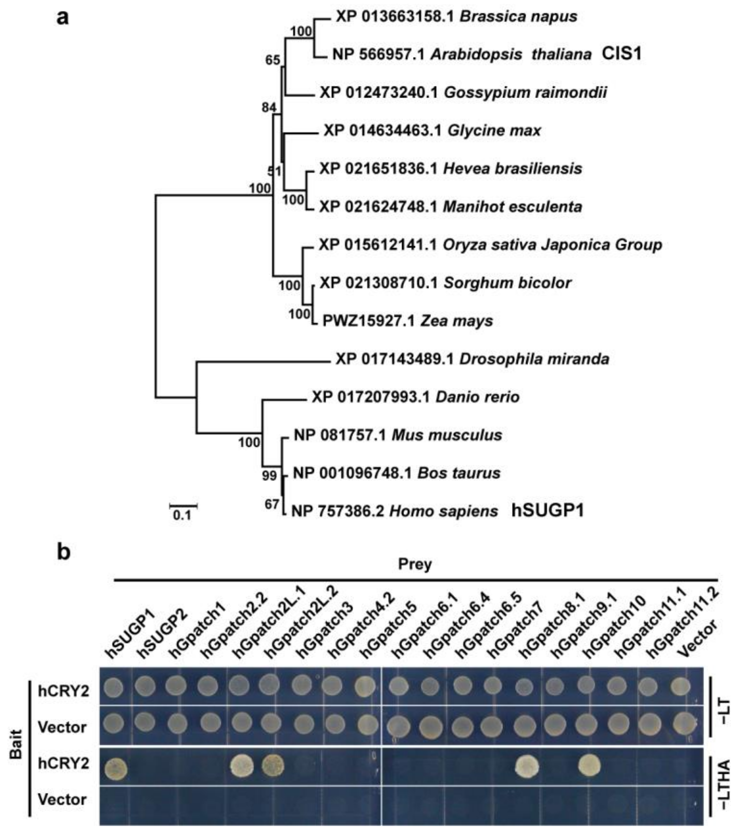

Fig. S2. Human CRY2 interacts with human CIS1 homologues.

(**a**), Phylogenetic tree of CIS1 homologues between animals and plants. Distances were estimated using the neighbor-joining algorithm. The numbers at the nodes represent the percentage of 1000 bootstraps. The scale bar indicates the average number of amino acid substitutions per site. (**b**), Histidine auxotrophy assays showing the interaction between hGpatchs and hCRY2.

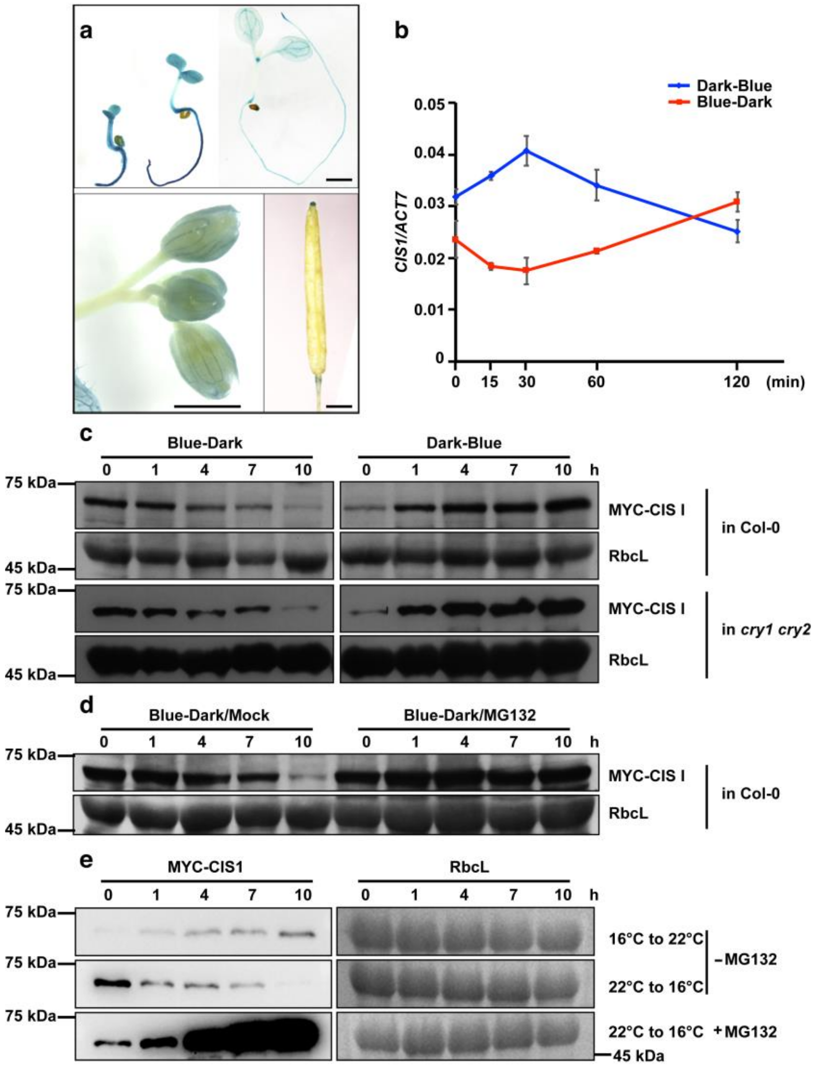

Fig. S3. Expression pattern of CIS1 in Arabidopsis.

(**a**), GUS-stained seedlings, inflorescence and silique of adult transgenic plants expressing the *pCIS1::GUS* transgene, scale bar = 1 mm. (**b**), Blue-light treatment affects the transcription of *CIS1*. Quantitative RT-PCR results showing the transcription level of *CIS1* is elevated for a limited time in response to blue-light treatment. 7-day-old Col-0 seedlings grown at 22°C in LD conditions were first treated with blue light (Blue) for 24 hours, and then transferred to dark (Dark). Alternatively, 7-day-old plants were first treated with dark (Dark) for 24 hours, and then transferred to blue light (Blue) for the indicated time. Error bars represent s.d. of three biological replicates. (**c**), CIS1 protein is degraded in the absence of blue light. Transgenic plants expressing *35S::Myc-CIS1* in the WT (Col-0) or *cry1 cry2* mutant background were grown in long days for 7 days, transferred to blue light (Blue) for 24 hours, and then transferred to dark (Dark) for the indicated time before sample collection. Alternatively, the 7-day-old plants were first transferred to dark (Dark) for 24 hours, and then transferred to blue light (Blue) for the indicated time. Rubisco (RbcL) is loading control. The immunoblot shows CIS1 degradation in darkness and accumulation when plants were moved to blue light. (**d**), The degradation of CIS1 in darkness is inhibited by the proteasome inhibitor MG132. Plants expressing *35S::Myc-CIS1* were grown in long day (LD)

conditions for 7 days, and leaves were excised and incubated with MG132 (50 μmol/L) or mock solution (0.1% DMSO) in darkness for the indicated time. Rubisco is shown as the loading control. (**e**), High ambient temperature stabilizes CIS1 protein. Transgenic plants expressing *35S::Myc-CIS1* were grown at 22°C in continuous white light for 5 days, moved to 16°C for 48 hours, and then transferred to 22°C for the indicated time. Alternatively, the 5-day-old plants were first moved to 22°C for 48 hours, and then transferred to 16°C for the indicated time. Rubisco is shown as the loading control. The immunoblot shows CIS1 protein degradation in low temperature and accumulation in higher temperature. The degradation of CIS1 in lower ambient temperature could be inhibited by the proteasome inhibitor MG132. In c, d, e, three independent experiments were performed with similar results.

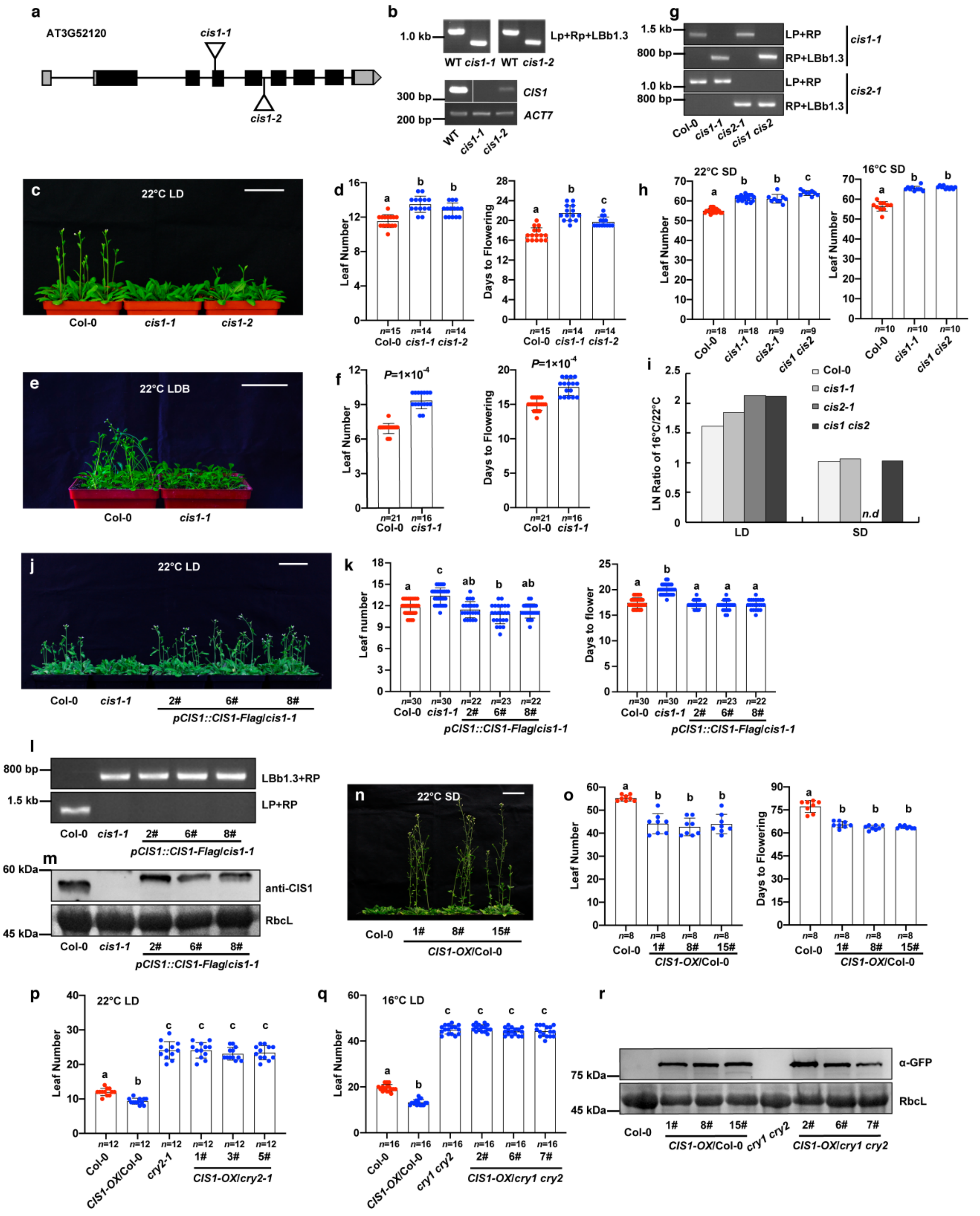

Fig. S4. CIS1 and CIS2 promote floral initiation.

(**a**), A diagram illustrating the genomic structure of *CIS1* and the locations of the T-DNA insertions. (**b**), PCR results showing the genotyping of the indicated mutant, and RT-PCR results showing

mRNA expression of *CIS1* in the genotypes indicated. (**c**, **e**), *cis1-1* mutants and Col-0 were grown in LD for 24 days (**c**) and in long-day blue light (LDB) for 18 days (**e**) when the photograph was taken. (**d**, **f**), Number of rosette leaves at the time of flowering and days to flowering for the indicated genotypes shown in **d** (for **c**), Number of total leaves at the time of flowering and days to flowering for the indicated genotypes shown in **f** (for **e**), the actual values were used to plot the graph. The standard deviations (s.d.) are shown. lowercase letters indicate statistically significant differences, as determined by one-way ANOVA with Tukey's multiple comparisons test ($P < 0.05$) in **d** and the asterisks represent a significant difference from Col-0 based on two sided Student's *t*-test in **f**. (**g**), PCR results showing the genotyping of the indicated genotypes. (**h**), Number of rosette leaves at the time of flowering for the indicated genotypes grown at 22°C and 16°C in SD conditions, the actual values were used to plot the graph. The s.d. are shown. lowercase letters indicate statistically significant differences, as determined by one-way ANOVA with Tukey's multiple comparisons test ($P < 0.05$). (**i**), Leaf number ratio of 16°C/22°C for indicated genotypes at LD and SD conditions. *n.d*, *not detected*. (**j**), 23-day-old WT, *cis1-1,* and three independent functional complementation transgenic lines expressing *pCIS1::CIS1-Flag* in the *cis1-1* mutant background grown at 22°C in LDs. Scale bar = 5 cm. (**k**), Number of rosette leaves at the time of flowering and days to flowering of the indicated genotypes shown in **j**, the actual values were used to plot the graph. The s.d. are shown. lowercase letters indicate statistically significant differences, as determined by one-way ANOVA with Tukey's multiple comparisons test ($P < 0.05$). (**l**), PCR results showing the genotyping of the indicated genotypes. (**m**), Immunoblot showing the CIS1 protein level in the genotypes indicated. RbcL serves as the loading control. (**n**), Flowering phenotype of CIS1 transgenic lines in SD. Three independent overexpression lines expressing *35S::CIS1* and the WT (Col-0) were grown in SD for 80 days when the photograph was taken. (**o**), Quantitative flowering time measured as the number of rosette leaves at the time of flowering and days to flowering of the indicated genotypes shown in **n**, the actual values were used to plot the graph. s.d. are shown. lowercase letters indicate statistically significant differences, as determined by one-way ANOVA with Tukey's multiple comparisons test ($P < 0.05$). (**p, q**), Quantitative flowering time measured as the number of rosette leaves at the time of flowering of the indicated genotypes, the actual values were used to plot the graph. s.d. are shown. lowercase letters indicate statistically significant differences, as determined by one-way ANOVA with Tukey's multiple comparisons test ($P < 0.05$). **(r),** Immunoblots showing the CIS1 protein level in *35S::YFP-CIS1* transgenic lines in WT (Col-0) and *cry1 cry2* backgrounds as indicated. RbcL serves as the loading control. In b, g, l, m, r, three independent experiments were performed with similar results.

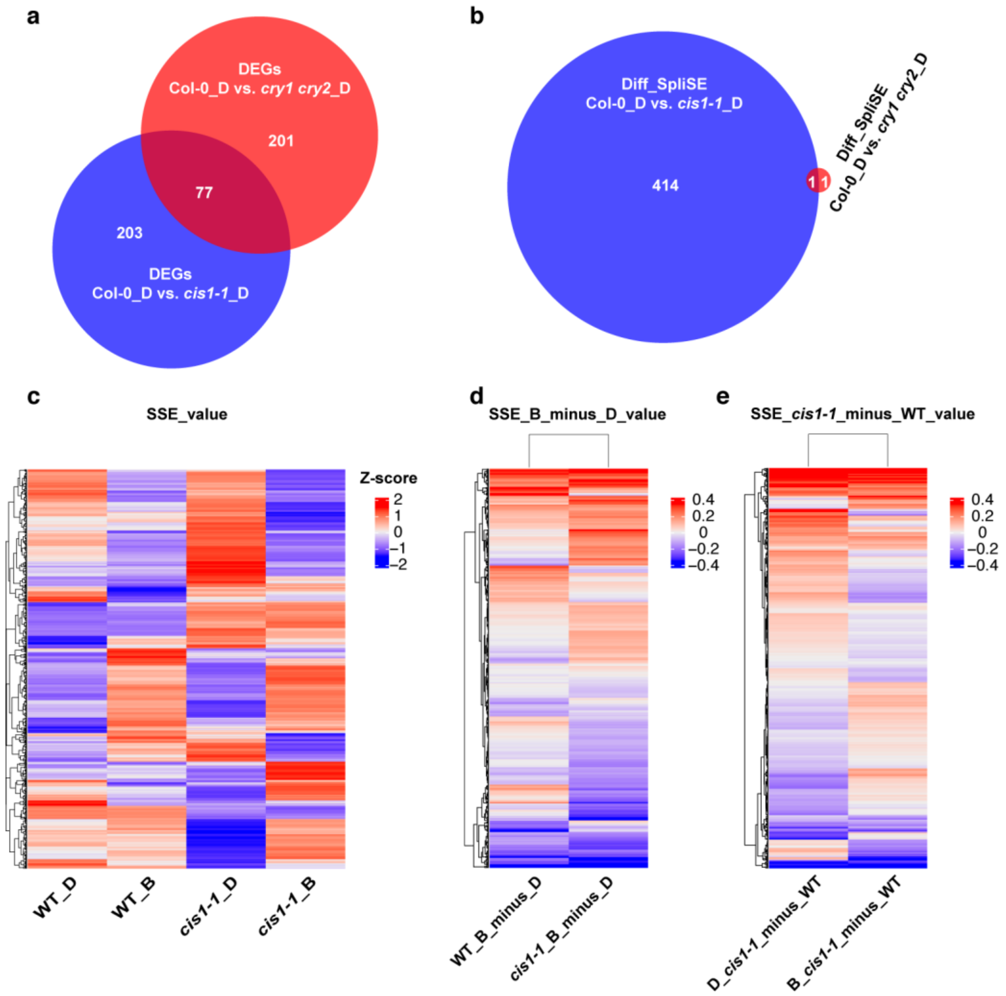

Fig. S5. Blue light and CRYs modulate RNA splicing via CIS1.

(**a**, **b**), Venn diagram showing the overlap between sets of differentially expressed genes (DEGs in **a**) and of differentially SpliSE genes (Diff_SpliSE in **b**) in Col-0 versus the *cry1 cry2* mutant in dark (Col-0_D vs. *cry1 cry2*_D) and Col-0 versus the *cis1-1* mutant in dark (Col-0_D vs. *cis1-1*_D). (**c**), Heatmap of pre-mRNA splicing profiles, quantified based on Z-scores of SSE values of Col-0 and *cis1-1* in the dark and Blue light (30 μmol·m^{-2}·s^{-1} for 3 hours). (**d**, **e**), Heatmap showing the effects of Blue light on splicing efficiency depends on CIS1 or not (**d**), or the effects of CIS1 on splicing efficiency depends on Blue light or not (**e**).

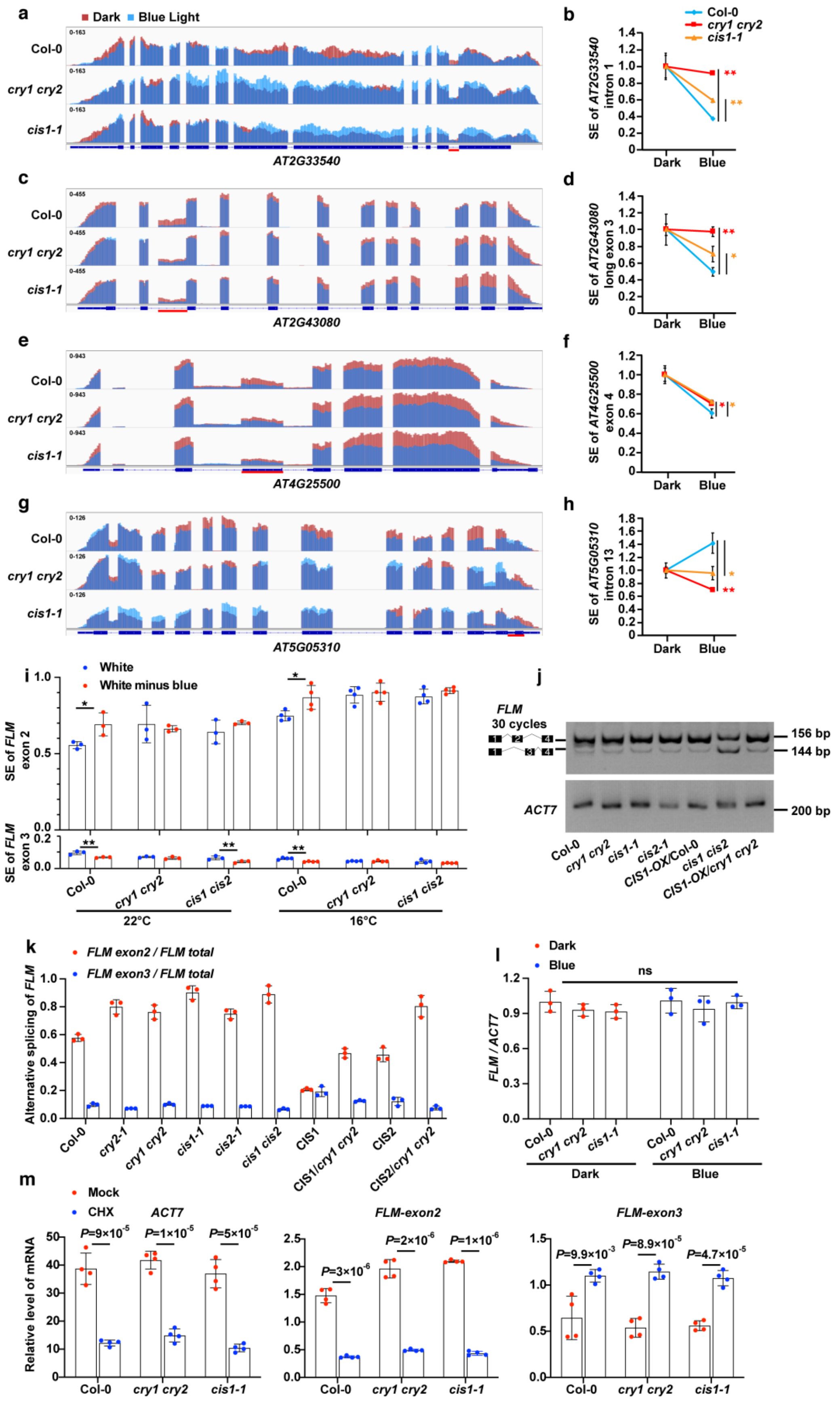
a
Dark Blue Light
Col-0
cry1 cry2
cis1-1
AT2G33540
b
Col-0
cry1 cry2
cis1-1
SE of AT2G33540 intron 1
Dark
Blue
c
Col-0
cry1 cry2
cis1-1
AT2G43080
d
SE of AT2G43080 long exon 3
Dark
Blue
e
Col-0
cry1 cry2
cis1-1
AT4G25500
f
SE of AT4G25500 exon 4
Dark
Blue
g
Col-0
cry1 cry2
cis1-1
AT5G05310
h
SE of AT5G05310 intron 13
Dark
Blue
i
White
White minus blue
SE of FLM exon 2
SE of FLM exon 3
Col-0
cry1 cry2
cis1 cis2
22°C
16°C
j
FLM
30 cycles
156 bp
144 bp
ACT7
200 bp
Col-0
cry1 cry2
cis1-1
cis2-1
CIS1-OX/Col-0
cis1 cis2
CIS1-OX/cry1 cry2
k
FLM exon2 / FLM total
FLM exon3 / FLM total
Alternative splicing of FLM
Col-0
cry2-1
cry1 cry2
cis1-1
cis2-1
cis1 cis2
CIS1
CIS1/cry1 cry2
CIS2
CIS2/cry1 cry2
l
Dark
Blue
ns
FLM / ACT7
Col-0
cry1 cry2
cis1-1
Dark
Blue
m
Mock
CHX
ACT7
FLM-exon2
FLM-exon3
Relative level of mRNA
P=9×10-5
P=1×10-5
P=5×10-5
P=3×10-6
P=2×10-6
P=1×10-6
P=9.9×10-3
P=8.9×10-5
P=4.7×10-5
Col-0
cry1 cry2
cis1-1

Fig. S6. CRYs and CIS1 mediate blue light-regulated RNA splicing and *FLM* alternative splicing.

(**a**, **c**, **e** and **g**), Integrative Genomics Viewer showing different AS events between Col-0, *cry1 cry2* and *cis1-1* detected by RNA-seq. Dark red (Dark) and blue (Blue light, 30 μmol·m^{-2}·s^{-1} for 3 hours) bars represent three biological replicates. Red lines in the gene schematic indicate the position of AS events in the genes. (**b**, **d**, **f** and **h**), RT-qPCR validation of RNA-seq data. Blue light-regulated splicing efficiency (SE) was calculated as the proportion of specific isoforms in among the corresponding total transcripts, normalized to that of the dark sample. Error bars represent the s.d. of three biological replicates. The asterisks indicate a significant difference from Col-0 based on two-sided Student's *t*-test (**P* < 0.05, ***P* < 0.01). (**i**), RT-qPCR showing blue light–regulated splicing of *FLM* in 10-day-old seedlings grown in 22°C and 16°C White light or White light with Cutoff filters to filter out all blue light (400 nm to 500 nm) LD conditions. Blue light–regulated splicing efficiency (SE) of *FLM* exon 2 and *FLM* exon 3 was calculated as proportion to total *FLM* transcripts. Error bars represent the s.d. of at least three biological replicates. The asterisks indicate a significant difference determined by two-way ANOVA with Šídák's multiple comparisons test. (**P* < 0.05, ***P* < 0.01). (**j**), RT-PCR showing the AS pattern of *FLM* transcripts over exon 1–exon 2 and exon 1–exon 3 of the indicated genotypes using 10-day-old seedlings grown at 16°C in LD condition. In j, three independent experiments were performed with similar results. (**k**), RT-qPCR showing the different splicing efficiency of *FLM* (*FLM exon2* and *FLM exon3*) for the indicated genotypes at 22°C LD conditions. Error bars represent the s.d. of three biological replicates. (**l**), RT-qPCR results showing the relative expression of total *FLM* in the indicated genotypes grown in dark for 4 days or moved to blue light for 3 hours. Error bars represent the s.d. of three biological replicates. ns, no statistically significant differences as determined by one-way ANOVA with Tukey's multiple comparisons test. (**m**), RT-qPCR analysis with primers specific to *FLM exon2*, *FLM exon3* and *ACT7* (as control), 7-day-old LD grown Col-0, *cry1 cry2* and *cis1-1* seedlings were treated with or without cycloheximide (CHX, 20 μM). Error bars represent the s.d. of four biological replicates. Two-sided Student's t-test was used to calculate *P* values.

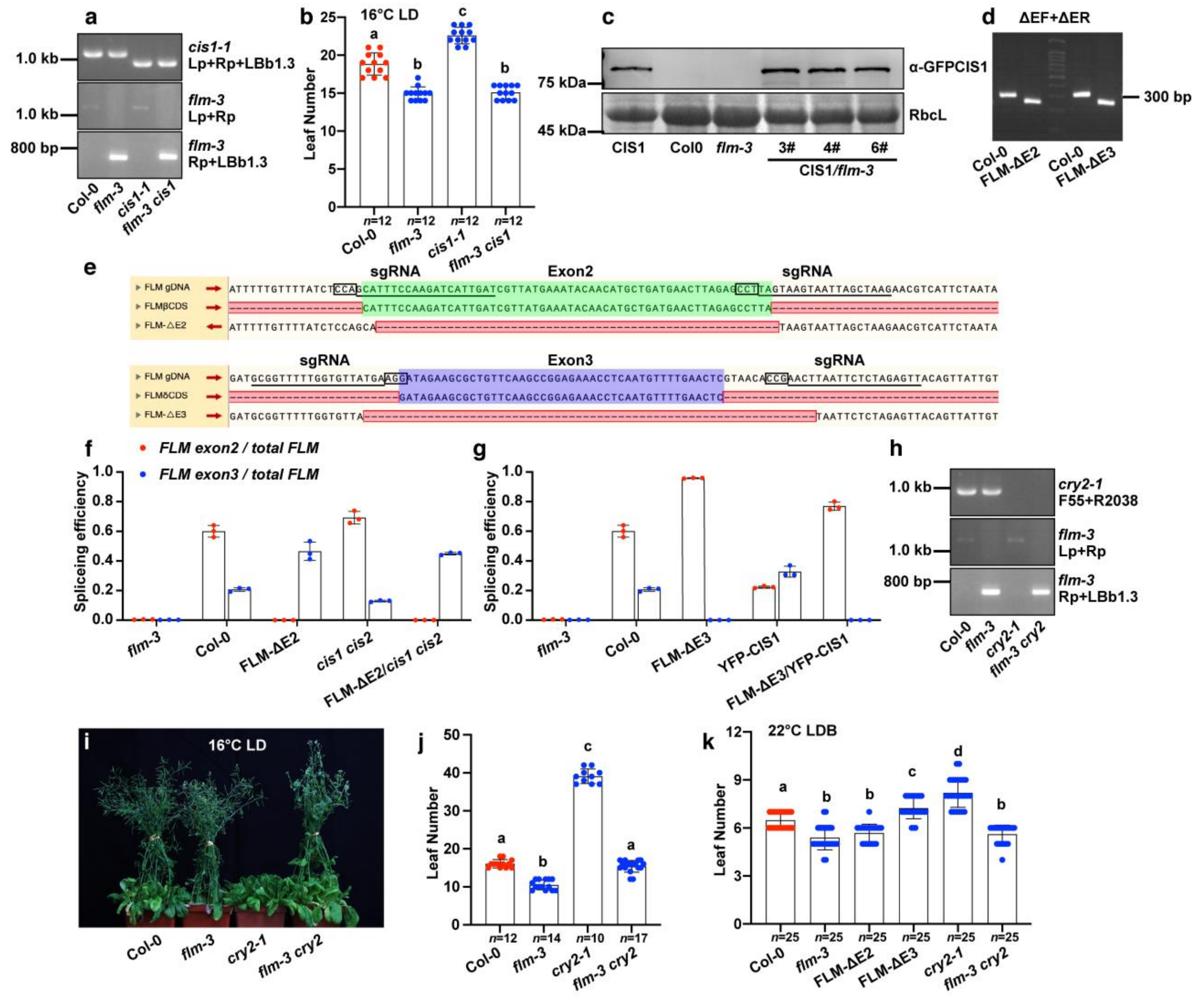

Fig. S7. CIS1 and CRY2 promotes flower initiation in a FLM-dependent manner.

(**a**), PCR results showing the genotyping of the indicated mutants used in Fig. 5a. (**b**), Number of total leaves at the time of flowering for the indicated genotypes grown in 16°C LD condition, the actual values were used to plot the graph. The s.d. are shown. Letters "a" to "c" indicate statistically significant differences, as determined by one-way ANOVA with Tukey's multiple comparisons test ($P < 0.05$). (**c**), Immunoblots showing the CIS1 protein level in the indicated genotypes used in Fig. 5c. (**d**), PCR results showing the genotyping of FLM-ΔE2 and FLM-ΔE3. (**e**), Sanger sequencing showing detailed positions of the deletions in the CRISPR lines FLM-ΔE2 and FLM-ΔE3. Position of exon 2 and exon 3 are green and purple, respectively. sgRNAs are underlined; PAM sites are indicated in boxes on the wild-type sequences. (**f**, **g**), RT-qPCR results showing the alternative splicing efficiency of *FLM* in the indicated genotypes used in Fig. 5e (for **f**) and Fig. 5f (for **g**). n.d., not detected. Error bars, s.d. of three biological replicates. (**h**), PCR results showing the genotyping of the indicated mutants. (**i**), 55-day-old plants of indicated genotypes grown in 16°C LD. Scale bar = 5 cm. (**j**), Number of rosette leaves at the time of flowering for the indicated genotypes shown in **i**, the actual values were used to plot the graph. The s.d. are shown. Letters "a" to "c" indicate statistically significant differences determined by one way ANOVA, Tukey's multiple comparisons test ($P < 0.05$). (**k**), Number of total leaves at the time of flowering for the

indicated genotypes grown in 22°C LD blue light (20 μmol m^{-2} s^{-1}) condition, the actual values were used to plot the graph. The s.d. are shown. Letters "a" to "d" indicate statistically significant differences, as determined by one-way ANOVA with Tukey's multiple comparisons test ($P < 0.05$). In a, c, d, h, three independent experiments were performed with similar results.

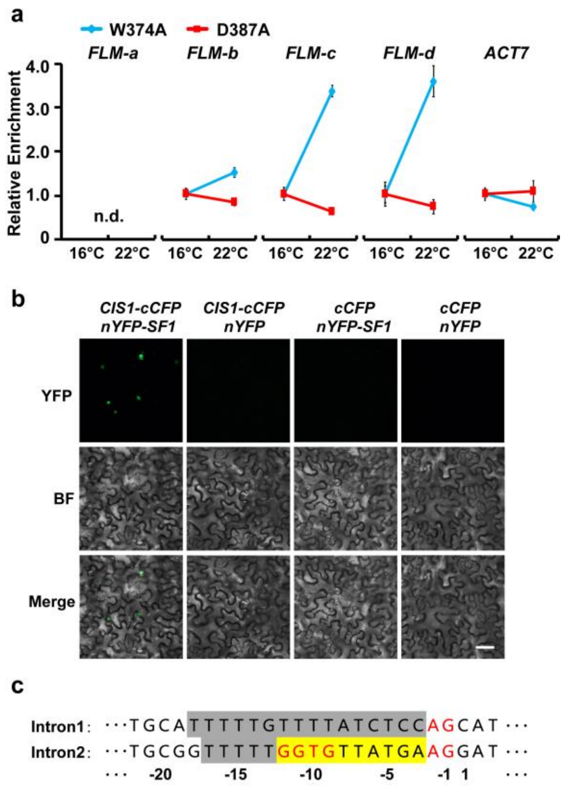

Fig. S8. CRY2 regulate the RNA binding activity of CIS1

(**a**), RIP-qPCR assay showing the binding affinity of CIS1 protein to *FLM* pre-mRNA *in vivo*. The 7-day-old 16°C continuous blue light (20 μmol·m^{-2}·s^{-1}) -grown CRY2^{W347A}/*cry1 cry2* and CRY2^{D387A}/*cry1 cry2* and *cis1-1* (mock) seedlings were transferred to 22°C continuous blue light (20 μmol·m^{-2}·s^{-1}) for 10 hours. RNA fragments (200–400 nt) extracted from seedlings were immunoprecipitated by the anti-CIS1 agarose beads (IP). The precipitated RNA was analyzed by RT-qPCR using different primer pairs of *FLM* pre-mRNA as indicated in Fig.6A. The *FLM* promotor and *ACT7* were analyzed as negative controls. The level of binding was calculated as the ratio between IP and mock, normalized to that of *IPP2* as an internal control, n.d., not detected. Error bars, s.d. of three biological replicates. (**b**), BiFC assay showing *in vivo* protein interactions between SF1 and CIS1. Leaf epidermal cells of *Nicotiana benthamiana* were co-transformed to express the fusion proteins as indicated. Scale bar = 20 μm. In b, three independent experiments were performed with similar results. (**c**), *Cis*-elements of a 3′ splice site (3′SS) comparison between intron1 and intron2 of *FLM*. The G-tract of intron2 (GGTG) between the Py and 3′AG is highlighted in yellow and with red font.
