# Supplementary material for: CRY2 interacts with CIS1 to regulate thermosensory flowering via FLM alternative splicing
Source: Nat Commun. 2022 Nov 17;13:7045. doi: 10.1038/s41467-022-34886-2 (PMC9671898; doi:10.1038/s41467-022-34886-2)
Supplement: Supplementary file 2 — Description of Additional Supplementary Files [file 41467_2022_34886_MOESM2_ESM.pdf]

Description of Additional Supplementary Files

File Name: Supplementary Data 1
Description: List of differentially expressed genes

File Name: Supplementary Data 2
Description: List of differentially spliced genes

File Name: Supplementary Data 3
Description: List of GO terms in the differentially spliced genes

File Name: Supplementary Data 4
Description: Genes showing AS in a blue light– and CIS1–dependent manner

File Name: Supplementary Data 5
Description: Different splice forms identified through Sanger sequencing

File Name: Supplementary Data 6
Description: Primers list
